# Australian native *Glycine clandestina* seed microbiota hosts a more diverse bacterial community than the domesticated soybean *Glycine max*

**DOI:** 10.1186/s40793-022-00452-y

**Published:** 2022-11-16

**Authors:** Ankush Chandel, Ross Mann, Jatinder Kaur, Ian Tannenbaum, Sally Norton, Jacqueline Edwards, German Spangenberg, Timothy Sawbridge

**Affiliations:** 1 Agriculture Victoria Research, AgriBio, Centre for AgriBioscience, Bundoora, VIC 3083 Australia; 2 School of Applied Systems Biology, La Trobe University, Bundoora, VIC 3083 Australia; 3 Agriculture Victoria Research, Australian Grains Genebank, Horsham, VIC 3400 Australia

**Keywords:** Seed bacterial microbiome, Soybean, *Glycine clandestine*, *Glycine max*, Core microbiome

## Abstract

**Background:**

Plant microbiome composition has been demonstrated to change during the domestication of wild plants and it is suggested that this has resulted in loss of plant beneficial microbes. Recently, the seed microbiome of native plants was demonstrated to harbour a more diverse microbiota and shared a common core microbiome with modern cultivars. In this study the composition of the seed-associated bacteria of *Glycine clandestina* is compared to seed-associated bacteria of *Glycine max* (soybean).

**Results:**

The seed microbiome of the native legume *Glycine clandestina* (crop wild relative; cwr) was more diverse than that of the domesticated *Glycine max* and was dominated by the bacterial class *Gammaproteobacteria*. Both the plant species (cwr *vs* domesticated) and individual seed accessions were identified as the main driver for this diversity and composition of the microbiota of all *Glycine* seed lots, with the effect of factor “plant species” exceeded that of “geographical location”. A core microbiome was identified between the two *Glycine* species. A high percentage of the *Glycine* microbiome was unculturable [*G. clandestina* (80.8%) and *G. max* (75.5%)] with only bacteria of a high relative abundance being culturable under the conditions of this study.

**Conclusion:**

Our results provided novel insights into the structure and diversity of the native *Glycine clandestina* seed microbiome and how it compares to that of the domesticated crop *Glycine max*. Beyond that, it also increased our knowledge of the key microbial taxa associated with the core *Glycine* spp. microbiome, both wild and domesticated. The investigation of this commonality and diversity is a valuable and essential tool in understanding the use of native *Glycine* spp. for the discovery of new microbes that would be of benefit to domesticated *Glycine max* cultivars or any other economically important crops. This study has isolated microbes from a crop wild relative that are now available for testing in *G. max* for beneficial phenotypes.

**Supplementary Information:**

The online version contains supplementary material available at 10.1186/s40793-022-00452-y.

## Background

The plant microbiome comprises a variety of microorganisms including bacteria, fungi, viruses and archaea [1]. In the natural environment, beneficial relationships formed between plants and microorganisms can promote and enhance plant health under various environmental stresses, both biotic and abiotic [2]. Recently, plant-associated bacteria (both endophytes and epiphytes) have been studied extensively due to their ability to confer numerous benefits (as biofertilizers and bioprotectants) in domesticated crops [3]. Individual bacterial strains have been inoculated into crop plants (e.g., legumes) by direct application in soil or as a seed coat for more efficient nutrient uptake (e.g., nitrogen and phosphorous) [e.g., BASF (Vault^®^), BioAg, Novozymes (Jumpstart^®^), ABM (Excalibre™) and MycoGold™], disease resistance [e.g., BioAg, Novozymes (Trichobank™)], plant growth and thus increased productivity and yields [4–6].

Plant seed consists of a diverse microbiome belonging to both epiphytes and endophytes. The stable environment that exists beneath the seed coat facilitates the vertical transmission of plant-beneficial microbial communities to successive plant generations [7–9]. In fact, the seed-associated bacteria can play an important role in seed preservation, breaking seed dormancy, increasing or decreasing seed germination rates, increasing early plant vigour and provide biotic-abiotic stress protections [10–12]. Once adapted to the physiological changes induced by the environment, the seed microbiota can then colonize nascent seedling roots or shoots and the surrounding rhizosphere of the emerging plant during the complex process of seed germination [13, 14]. The seed-borne microbes can enhance seed germination and can also confer various benefits to host plants such as growth promotion and biocontrol activity against phytopathogens [15–23]. However, the majority of seed-associated bacteria have proven to be difficult to isolate by conventional culturing techniques, and thus far, have not been studied extensively because of this limitation [24].

Seeds are known to contain a core microbiome that is suggested to contain bacterial genera that are selected through a long-term selection process and are well adapted to the internal microhabitat of plant seeds [25]. For instance, *Pseudomonas*, *Pantoea*, *Methylobacterium*, *Bacillus*, *Sphingomonas*, *Curtobacterium* and *Microbacterium* are part of the core microbiome of numerous different plant species [7, 24, 26–29]. Previously, functions and composition of the core microbiome of many domesticated plants have also been successfully characterised (e.g., *Arabidopsis thaliana* L., *Glycine max*, *Hordeum vulgare* and *Oryza* spp.) [29–33].

Plant domestication known to cause compositional changes in plant microbiome that has resulted in loss of potentially-beneficial microbes associated with the many wild crop cultivars of maize, wheat, rice and the common bean [34]. Literature has also reported similar shifts in the seed microbiome composition of native species [7, 24, 28, 34, 35], although our understanding about the seed microbiome of native plants of economically important crops is still very limited. A better understanding of the native seed microbiome can help us to identify the key microbial components that are of significance for the survival of such plants under harsh biotic and abiotic conditions and, as such, these microbes can then be potentially re-integrated back into the domesticated seed microbiome to uptick productivity and plant protection. Thus, these ‘lost’ microbes must be thoroughly assessed for their application in sustainable agriculture practices for possible increased productivity and yields [24, 36, 37].

The common soybean (*Glycine max* L. Merr.) is a legume (*Fabaceae*) crop plant found in Africa, East Asia and Australia [38]. Soybean is one of the major sources of dietary protein and oil for both humans and livestock [39–41]. It is also a well-known leguminous species that can fix nitrogen in association with rhizobia [38, 42]. The *Glycine* subgenus comprises about 30 perennial species, of which 26 wild perennial species are native to Australia and widely distributed across different types of habitats [43, 44]. Studies have shown that *Glycine* cwr are genetically more diverse, and, when compared to the domesticated variety, perennial *Glycine* cwr outperformed the commercial soybean cultivar under multiple biotic and abiotic stress conditions [39]. Furthermore, another study found that the soybean cwr were able to form a fully-fledged symbiotic relationship with more diverse combinations of rhizobia strains than a newly-derived domesticated soybean cultivar, based on differences in their total final yield [45]. The knowledge about key *Glycine* cwr seed bacteria can be used to prepare several bacterial consortia to assess their potential benefits to *G. max* and other agriculture crops. This study aims to characterise the seed microbiome of individual *Glycine* cwr seed lots (accessions) in anticipation of improving the productivity of domesticated *G. max*.

To explore the seed bacterial microbiome of *Glycine* cwr and to identify the core microbiome associated with a native *Glycine* species, we selected *G. clandestina* from six different natural locations in the Greater Melbourne area of Victoria, Australia. In this study, a total of five seed accessions of Australian-grown and commercially available *G. max* (L.) Merr. were used for comparison of seed microbiota. We hypothesized that the bacteria present both in and on the *Glycine* cwr *G. clandestina* may be able to colonize the seedlings of *G. max* and provide useful functions to the crop. It is also known that surface sterilization will kill certain microbes residing in the outer seed compartment and may eliminate some essential bacteria [46]. It was decided not to differentiate between the epiphytic and endophytic bacteria populations, and we determined the complete seed microbiome profile by assessing non-surface sterilized seeds (washed with sterile water only) through amplifying the V3 to V4 region in the 16S rRNA gene of the bacteria. These seeds were also assessed by isolating seed microbes from germinated seedlings to examine the culturable microbiome.

## Materials and methods

### *Glycine* seed collection

Seed pods of *Glycine clandestina* were collected from six different “Seed Accessions” across greater Melbourne, Victoria, identified by using the online database “The Atlas of Living Australia” https://bie.ala.org.au/search?q=Glycine+clandestina (accessed on 22 January 2020). The seed pod collections were performed between November 2018 and January 2019 at the time of pod maturation stage, where the pod colour turns a dark brown (Table 1). There was a minimum 15 km distance between each seed accession and pods were collected from an individual plant in the identified area. Seed pods were collected into a paper bag while wearing gloves and allowed to dry and shatter naturally on a benchtop in glasshouse conditions (Light: 22 °C for 14 h and Dark: 14 °C for 10 h). Seeds for the Australian-grown soybean cultivar (*G. max*) were obtained from Australian Grains Genebank, Horsham and three other commercial seed suppliers from NSW and QLD, Australia (Table 1).

**Table 1:** Description of *Glycine* seeds

Seed type	Species	Seed accession/geographical location	Year of collection	Number of replicates processed
Crop wild relative seed	*G. clandestine*—Butterfield Wildlife Reserve	(− 37.8969952778, 145.441033611)	2018	16
	*G. clandestina*—Cardinia Creek	(− 37.7977694444, 145.451086667)		15
	*G. clandestina*—Wandin Yallock Creek Reserve	(− 37.97448333, 145.39268694)		16
	*G. clandestina*—Running Creek Road	(− 37.5401, 145.202)		9
	*G. clandestina*—Dandenong Ranges National Park	(− 37.8809083, 145.3163306)	2019	10
	*G. clandestina*—Mornington Peninsula National Park	(− 38.422287, 144.95559200000002)		7
Domesticated crop seed	*G. max*—AGG Batch-1 (69,615 SOYB 1)	AGG	2018	8
	*G. max*—AGG Batch 2 (Burrinjuck)	AGG	2019	13
	*G. max*—Green Harvest (Endamame)	Green Harvest, QLD		4
	*G. max*—Wholesome Supplies	Wholesome Supplies, QLD		7
	*G. max*—Seed Grass	Australian Wheatgrass, NSW		8

Once the seed pods dried and shattered, seeds were then stored under room temperature conditions in a ziplock bag. For seed microbiome profiling, only germinated seeds were selected. In this study, about 4–16 seedling replicates were processed per accession (each seedling was considered as a biological replicate) (Table 1).

### Seed germination

*G. max* and *G. clandestina* (cwr) seeds were washed ten times with an excess of sterile distilled water under sterile conditions. All cwr seed surfaces were then scarified using a sterile scalpel blade to initiate water imbibition. Seeds of both *G. max* and the cwr were germinated in large (12-cm diameter) sterile petri dishes by placing seeds between Whatman™ paper soaked in sterile distilled water (two papers under and one on top of the seeds). The petri dishes were then sealed with Parafilm™ and incubated for 48–72 h in darkness at room temperature. After the dark incubation, the top layer of filter paper was removed under sterile conditions and the petri dishes were resealed with Parafilm™. There followed a further 9–11 days of incubation on a lab benchtop under ambient light conditions. If needed, sterile water was sprayed on seedlings during the incubation under sterile conditions for adequate hydration. Seedlings were harvested for microbiome analysis once the cotyledons reached the un-folded growth stage (Additional file 1: Fig. S2A, B).

### Microbiome profiling

#### Microbial DNA extraction and amplicon library construction

For seed microbiome profiling (*G. clandestina* and *G. max*), 8–16 seedlings that reached the unfolded stage were selected for each accession. Whole seedlings (root, shoot and cotyledon) were cut into pieces of approximately 0.5–1 cm a using sterile scalpel blade, collected in 1.2 mL QIAGEN collection tubes and snap-frozen in liquid nitrogen and stored at − 80 °C until being processed for DNA extraction. DNA extraction was performed using the MagAttract^®^ 96 DNA plant kit using a Biomek FX^P^ Lab Automation Workstation coupled to a Synergy 2 multi-mode reader controlled by Biomek software version 4.1 and Gen 5 (2.08) software (Biotek Instruments, USA) with slight changes in manufacture’s guidelines.

Amplicon libraries for Illumina sequencing were prepared using barcoded primer 5151f-806r, specific to V4-region of the bacterial 16s rRNA gene. Amplification of the host chloroplast and mitochondrial 16s DNA was blocked by adding peptide nucleic acid, pPNA and mPNA respectively to the PCR mix. PCR for 16s rRNA gene amplification was performed in a total volume of 25 µL Kapa HiFi Hotstart 2 × ReadyMix DNA polymerase (Kapa Biosystems Ltd., London, UK), 50 µM of pPNA and mPNA mix, 5 µM of each primer, PCR grade water, and 5 µL of template DNA) under the following cycling conditions: 94 °C for 3 min., 30 cycles of 94 °C for 15 s, 75 °C for 10 s, 55 °C for 10 s, 72 °C for 45 s, and a final elongation at 72 °C for 10 min. Libraries were further purified using AMPure XP beads (LABPLAN; Naas, Ireland). Dual indices and Illumina sequencing adapters from the Illumina Nextera XT index kits v2 B and C (Illumina, San Diego, USA) were added to the target amplicons in a second PCR step using Kapa HotStart HiFi 2 × ReadyMix DNA polymerase (Kapa Biosystems Ltd., London, UK). Cycle conditions were 95 °C for 3 min, then 10 cycles of 95 °C for 30 s, 55 °C for 30 s, 72 °C for 30 s, then a final extension of 72 °C for 5 min. followed by library clean up using AMPure XP beads.

The barcoded libraries were quantified on a Nanodrop™ 1000 spectrophotometer and pooled together in an equimolar concentration. Library pools were further quantified for concentration and size using QuantiFluor^®^ dsDNA assay (Promega Corporation, USA) and a Tape station 2200 High Sensitivity D1000 kit (Agilent Technologies, USA) respectively. Paired-end sequencing was performed on an Illumina Hiseq 3000 amplicon sequencing using a 2 × 150 bp v3 chemistry cartridge, except some samples were sequenced on a Miseq v3 (2 × 300 bp v3 chemistry cartridge). All Illumina sequences have been submitted to the NCBI short read Archive (SRA accession PRJNA811248).

#### Bioinformatic analysis of 16s rRNA gene amplicon library sequences

The raw Illumina-paired end reads were trimmed, quality-filtered and merged into a single read length of 253 bp using PANDAseq with the following overlap threshold: -o 150 -O 300 (for Miseq data: 2 × 300 bp) and -o 8 -O 8 (for Hiseq 3000 data: 2 × 150 bp) [47]. Afterwards, sequencing data analysis was performed using QIIME 2 2020.2 following pipeline in “Moving Pictures” tutorials [48]. The DEBLUR algorithm was applied to filter the chimeric reads and to obtain a feature table containing the amplicon sequencing variants (ASVs) and representative sequences [49]. The ASVs were further aligned with mafft [50] via q2-alignment and then used to construct a phylogeny tree with fasttree2 [51] via q2-phylogeny. The ASVs were then taxonomically classified using a naïve Bayes taxonomy classifier [52] trained on the silva-132 release (V4 region-16s rRNA gene) [53]. Plant associated reads (mitochondria and chloroplast) and low abundance features (minimum 10 counts per feature that were present in at least 2 replicates) were removed from the data using the filter-features plugin.

Alpha- (Shannon diversity) and β-diversity (Unweighed UniFrac distances) between and within *G. max* and *G. clandestina* accessions was explored running the core-metrics script in QIIME2 by rarefying feature table to the lowest value of read counts (1600 sequences) present in one sample. Core bacterial features (features present in > 95% the samples) at the genus level were also identified within and across *G. clandestina* and *G. max* accessions. Venn diagrams were plotted in Genedata Expressionist^®^ Analyst™ v.10.0 (Genedata; Basel, Switzerland) by exporting the grouped rarefied feature table to determine the core features. Representative sequences were exported and mapped against the assembled genomes of bacterial isolates isolated from all accessions of *G. clandestina* to assess which 16S rRNA gene sequences matched cultivated isolates.

Statistical analyses of the 16s rRNA gene data was performed using scripts in QIIME2 2020.2. Alpha-diversity was tested for significant differences using the Kruskal–Wallis pairwise test and β-diversity using the permutational multivariate analysis of variance (PERMANOVA) and PERMDISP test using default parameters. Mantel test was performed for *G. clandestina* seed data to evaluate correlations between community structure based on Unweighed UniFrac distances and Seed accession/Geographical location using q2-coordinattes plugin in QIIME2. Mantel test was not performed for *G. max* seed accession as geographical location data was not available.

### Microbial isolation

#### Isolation of *Glycine* seed-associated bacteria

After 10 or 11 days of growing on moistened filter paper, seedlings of *G. max* (one accession) and *G. clandestina* (six accessions) were harvested in triplicate (one seedling per replicate) by removing the shoot and root tissues and discarding the seed coat. Plant tissues were cut in small pieces (0.5–1 cm) and homogenized either using a sterile pestle or two cycles of a Qiagen TissueLyser II for one minute at 30 Hertz in 200–400 µL of 1 × PBS buffer. The resulting macerates were serially diluted (10^−1^–10^−4^) and a 20 µL aliquot was plated onto Reasoner’s 2A agar (R2A, Oxoid, UK) and incubated at room temperature for up to four weeks. Colonies of different morphologies were picked and subcultured onto fresh R2A plates. Some isolates could be obtained from the 10^−4^ dilutions, however most originated from the 10^0^–10^−3^ dilutions. Pure subcultures were further grown in Reasoner’s 2A Broth (R2B) for 24–48 h and stored in 20% glycerol at –80 °C.

#### Rapid microbial identification by MALDI-TOF MS analysis

##### Preparation of sample for MALDI-TOF MS analysis

MALDI spectra were acquired for all isolated unknown bacterial strains to determine the similarity between each strain. The protein profiles of each bacterial strains were acquired for analysis using the Bruker MALDI BioTyper system. Single colonies of bacterial strains were obtained by streaking from glycerol stock or from freshly cultured colonies onto R2A plates after allowing colony growth for 24–48 h at room temperature. The formic acid extraction method was used to obtain MALDI spectra according to the manufacturer’s instructions. A small quantity (0.1–0.5 mg) from a single bacterial colony was directly transferred to a 384-ground steel MALDI target plate in duplicate and air-dried at room temperature. The dried cells were then overlaid with one µL of 70% formic acid, gently mixed by pipetting and air-dried, followed by adding 1 µL of matrix solution [α-cyano-4-hydroxycinnamic acid (10 mg HCAA in one mL of solvent solution: 50% volume μL ACN (acetonitrile), 47.5% volume μL water, and 2.5% volume μL TFA (trifluoroacetic acid))]. The plate was then dried at room temperature. *Escherichia coli* strain ATCC 25922 was included as a biological quality control. The target plate was analysed in a Bruker MALDI-TOF ultraflextreme mass spectrometer (Bruker Daltonics, Germany) coupled with Flex Control 3.3 software (Bruker Daltonics, Germany) according to the manufacturer’s protocol. Protein spectra were calibrated with the *Escherichia coli* ATCC 25,922 quality control strain as an internal bacterial test standard (Bruker Daltonics, Germany).

All protein spectra measurements were performed automatically using Flex Control software with following set-up values in the linear positive mode: ion source 1 voltage, 25.01 kV; ion source 2 voltage, 23.22 kV; lens voltage. 7 kV; mass range, 2–20 kDa. The final spectra was the sum of eight single spectra, each obtained by 200 laser shots on random target spot positions.

##### Bacterial classification and identification

Protein spectra were compared to the MALDI BioTyper library (3,746 spectra as of June 9, 2010), which included an in-house endophyte library, for preliminary identification and taxonomical assignment using a Bruker BioTyper 3.1 real-time classification software (Bruker Daltonics, Germany). The following score values were assigned by MALDI Biotyper classification results: < 1.7 (unreliable classification); 1.7–2.0 (genus identification); 2.0–2.3 (probable species identification) and 2.3–3.0 (exact species identification).

##### MALDI-TOF MS spectra analysis

The raw protein spectra from each plate were processed separately through a data deconvolution workflow in the Genedata Expressionist^®^ Refiner MS™ v.10.0 (Genedata; Basel, Switzerland). First, spectra were aligned using a m/z grid window of ten scans followed by a baseline subtraction to reduce background noise across the grid at 20% quantile with m/z window of 25 Da and finally performing m/z alignment with the reference spectra (i.e., *E. coli* ATCC 25922) using average spectrum method with m/z window of 100 Da and m/z shift range of 2–4 Da; spectrum. Spectra were then merged and processed further, first by repeating the alignment m/z across reference spectra (*E. coli* ATCC 25922) from the grid, followed by spectrum smoothing with a m/z window of 5 points to reduce intensity jitter of putative peaks, followed by restricting the m/z range to 2000–15,000 Da, which is recommended by Bruker to capture all protein peaks. These spectrum peaks were then detected using a resolution-based method with standard detection and computing peak centre using local maximum and determining peak boundary using maximum curvature. Finally, the valid peaks were filtered with an intensity threshold 10% and a minimum presence threshold in two experiments.

Peak lists of individual spectra were converted into a matrix and exported to Genedata Expressionist^®^ Analyst™ v.10.0 (Genedata; Basel, Switzerland) for analysis. A hierarchical clustering analysis was conducted to compare the difference between the protein spectra of each bacterial isolate. The analysis utilized the positive correlation (1-r) distance algorithm, with complete linkage, and only included values present in 50% of samples. A Hierarchical Clustering tree was generated, whereby novel bacterial isolates were clustered based on similar protein profiles.

#### Microbial identification by whole genome sequencing

##### DNA extraction and library preparation

In total, 36 isolates representing different clades from the resultant MALDI Hierarchical Clustering tree were selected for genotyping (Additional file 2: Fig. S1). For DNA extraction, all isolates were incubated overnight in 50 mL of Reasoner’s 2A Broth (R2B) at 26 °C in a shaking incubator (set at 190 rpm). At the end of the incubation period, bacterial cultures were processed for DNA extraction using the Promega™ Wizard™ Genomic DNA Purification Kit (USA), according to the manufacturer’s guidelines. Optical density measurements of the gDNA were performed in a Quantus™ Fluorometer (Promega Corporation, Madison, Wisconsin, United States).

Libraries were prepared from 1 ng of input gDNA by enzyme fragmentation and tagging with sequencing adapters using Nextera XT DNA Library Preparation kit (Illumina, San Diego, California, United States). Finally, libraries were quantified using the QuantiFluor^®^ dsDNA assay (Promega Corporation, USA) and Tape station 2200 High Sensitivity D1000 kit (Agilent Technologies, USA). Libraries were further pooled in an equimolar concentration and sequenced on an Illumina HiSeq 3000 amplicon sequencing using a 2 × 150 bp v3 chemistry cartridge.

##### Sequencing data analysis and mapping of 16S rRNA gene ASVs

The sequence data (raw reads) was assessed for quality and filtered to remove adapter and index sequence, and low-quality bases using fastp using following parameters: *-w 8 -3 -5* [54, 55]. De novo assembly of high-quality raw reads was performed with Unicycler (v0.4.8) [56]. The Bandage software was then used to evaluate the de novo assemblies by visualizing the assembly graphs [57]. Next, assembled genomes were taxonomically classified by Kraken2 [58] using a custom database containing all completed bacterial reference genomes in NCBI (20/03/2020) [58]. Furthermore, 16S rRNA metagenomic gene ASVs exported from QIIME2.2020.2 were then mapped to the bacterial sequences by creating an in-house BLAST database of the genome assemblies and aligning sequences using the BLASTn tool.

## Results

### Microbiome profiling

#### Exploring bacterial communities associated with the *Glycine* species

After paired-end alignments, quality filtering, removal of low frequency sequences (< 10 counts), singletons and chimeric sequences and plant sequences, a total of 21,193,644 sequences remained, split between *G. clandestina* (11,159,724) and *G. max* (10,033,920), respectively. These reads were then assigned to ASVs (Amplicon Sequence Variants) resulting in a table with 140 ASVs (*G. clandestina*) and 222 ASVs (*G. max*). The ASVs table was then rarefied to a sampling depth of 1600 bacterial sequences containing a total of 69 ASVs (*G. clandestina*) (Additional file 1: Table S6) and 45 ASVs (*G. max*) at the genus level, based on the lowest number of sequences per sample (Additional file 1: Table S7).

#### Identification of the key drivers of the *Glycine* bacterial microbiome

**Fig. 1:**
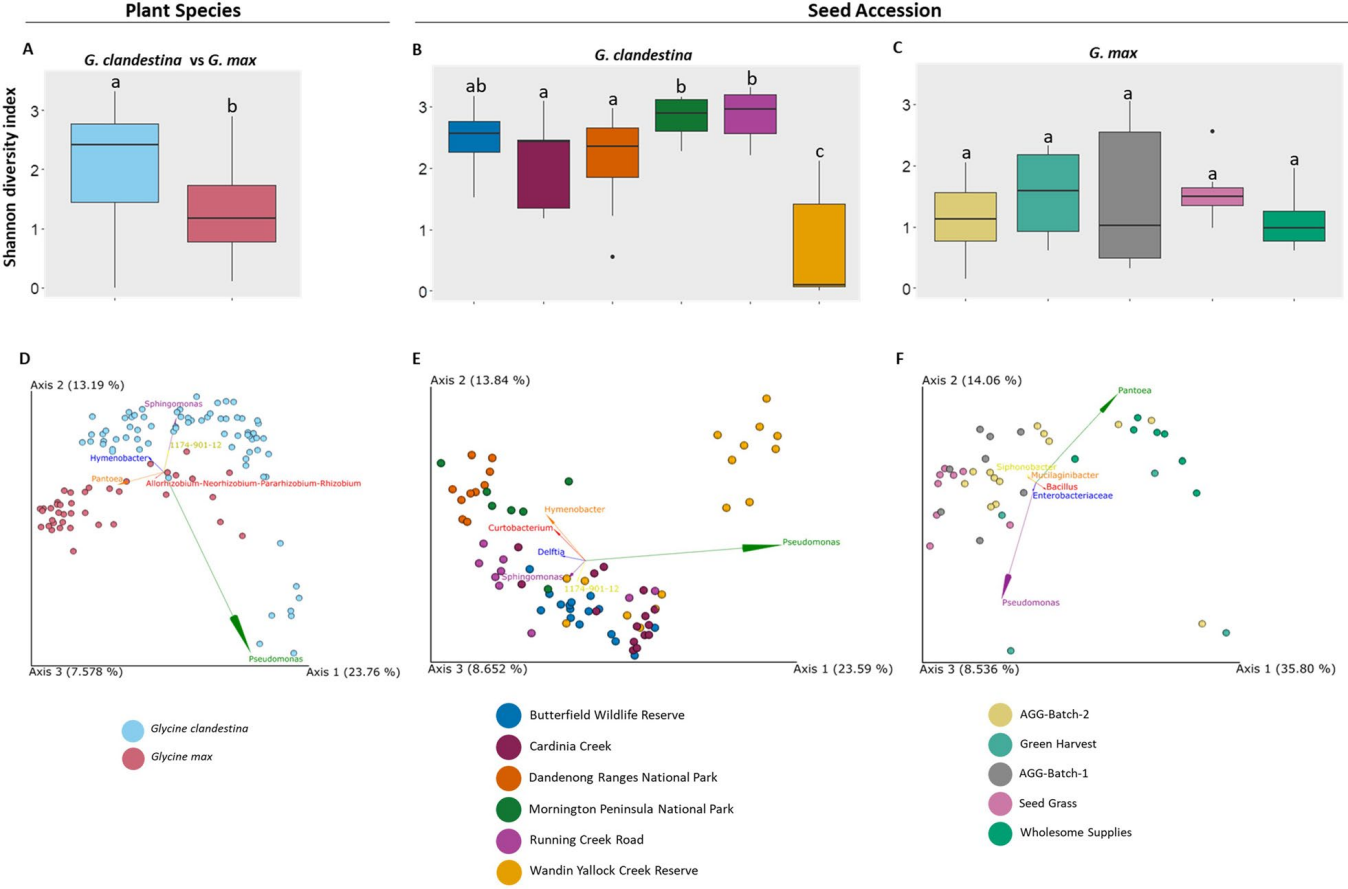
Alpha- (α-) and Beta (β)-diversity analyses of the seed microbiome of both *Glycine* species. Box-and-Whiskers-plots visualize the Shannon diversity index based on “Plant Species” (**A**) and “Seed Accession” (**B**, **C**). Significant differences (*p* ≤ 0.05) were assessed by the Kruskal Wallis pairwise test and are indicated by the lower-case letters. Community clustering of bacterial composition based on “Plant Species” (**D**) and “Seed Accession” (**E**, **F**) are indicated by two-dimensional unweighted-Unifrac distances PCoA biplots at genus level. Different colours of the data points represent different plant species (**A**, **D**) and seed accession (**B**, **C**, **E**, **E**). Significant differences in bacterial composition were tested using the PERMANOVA and PERMDISP test

The Shannon diversity index was used to assess the bacterial diversity within all *Glycine* seeds (Additional file 1: Table S2). The significant differences (*p* < 0.05) between seed accessions of both *Glycine* species were calculated using the non-parametric Kruskal–Wallis pairwise test (Additional file 1: Table S3). Samples were grouped as “Plant Species” and “Seed Accession” to identify the dependencies of microbial diversity on either category (Fig. 1A–C). Based on “Plant Species”, bacterial diversity within the *G. clandestina* (2.4) microbiome was found to be significantly more diverse than within *G. max* (1.2) (*p* = 0.000042). While based on “Seed Accession”, bacterial diversity was significantly different in the individual seed accessions of *G. clandestina.* Seed accessions from “Running Creek Road” (2.96), “Mornington Peninsula National Park” (2.89) and “Butterfield Wildlife Reserve” (2.56) had the greatest diversity, with “Cardinia Creek” (2.43) and the “Dandenong Ranges National Park” (2.35) having significantly lower diversity (except Butterfield Wildlife Reserve), whilst “Wandin Yallock Creek Reserve” (0.09) had significantly lower diversity than all other accessions (Fig. 1B). In the case of “Seed Accession” for *G. max,* no significant differences were observed within any of the seed accessions (Fig. 1C).

To evaluate the main driver of the *Glycine* seed bacterial microbiome composition, β-diversity analysis was conducted using an Unweighted UniFrac distance matrix PCoA (Fig. 1D–F) in combination with PERMANOVA and PERMDISP test (Additional file 1: Table S4). Based on “Plant Species”, *G. clandestina* formed a clear cluster separate from *G. max*, with some commonality (Fig. 1D). In addition, “Seed Accessions” formed distinct clusters for both *G. clandestina* and *G. max* (Fig. 1E, F). Interestingly, the seed accession “Wholesome Supplies” in *G. max* formed a totally separate cluster to the other four seed accessions. From the PERMANOVA test, significant differences in the bacterial microbiome composition were also observed for both “Plant Species” and “Seed Accession” (*p* < 0.05) (Additional file 1: Table S4). Results of PERMDISP test indicated that the dispersion was only significant between some *G. clandestina* (*p* < 0.05) seed accession including Butterfield Wildlife Reserve vs Dandenong Ranges National Park, Butterfield Wildlife Reserve vs Wandin Yallock Creek Reserve, Cardinia Creek vs Dandenong Ranges National Park, Cardinia Creek vs Wandin Yallock Creek Reserve,Dandenong Ranges National Park vs Mornington Peninsula National Park, Dandenong Ranges National Park vs Running Creek Road and Dandenong Ranges National Park vs Wandin Yallock Creek Reserve. While no significant differences were observed between all *G. max* seed accession (Additional file 1: Table S4). The non-significant PERMDISP results indicated that within group dispersions were homogeneous, therefore the results of the PEERMANOVA can be interpreted as true differences in composition of microbial communities. Interestingly, the PERMDISP results showed that bacterial communities were significantly (*p* < 0.05) dispersed between both *Glycine* species when data was grouped based on “Plant Species” (Additional file 1: Table S4).

Mantel test was performed to detect correlation between the *G. clandestina* microbiome composition and Seed accession/Geographical location. We found that the Seed accession/Geographical location was positively associated with the dissimilarity of *G. clandestina* seed microbiome (Mantel test, *r* = 0.44, *p* = 0.001). This correlation was relatively low, but significant.

#### Taxonomic classification of *Glycine* bacterial microbiome

**Fig. 2:**
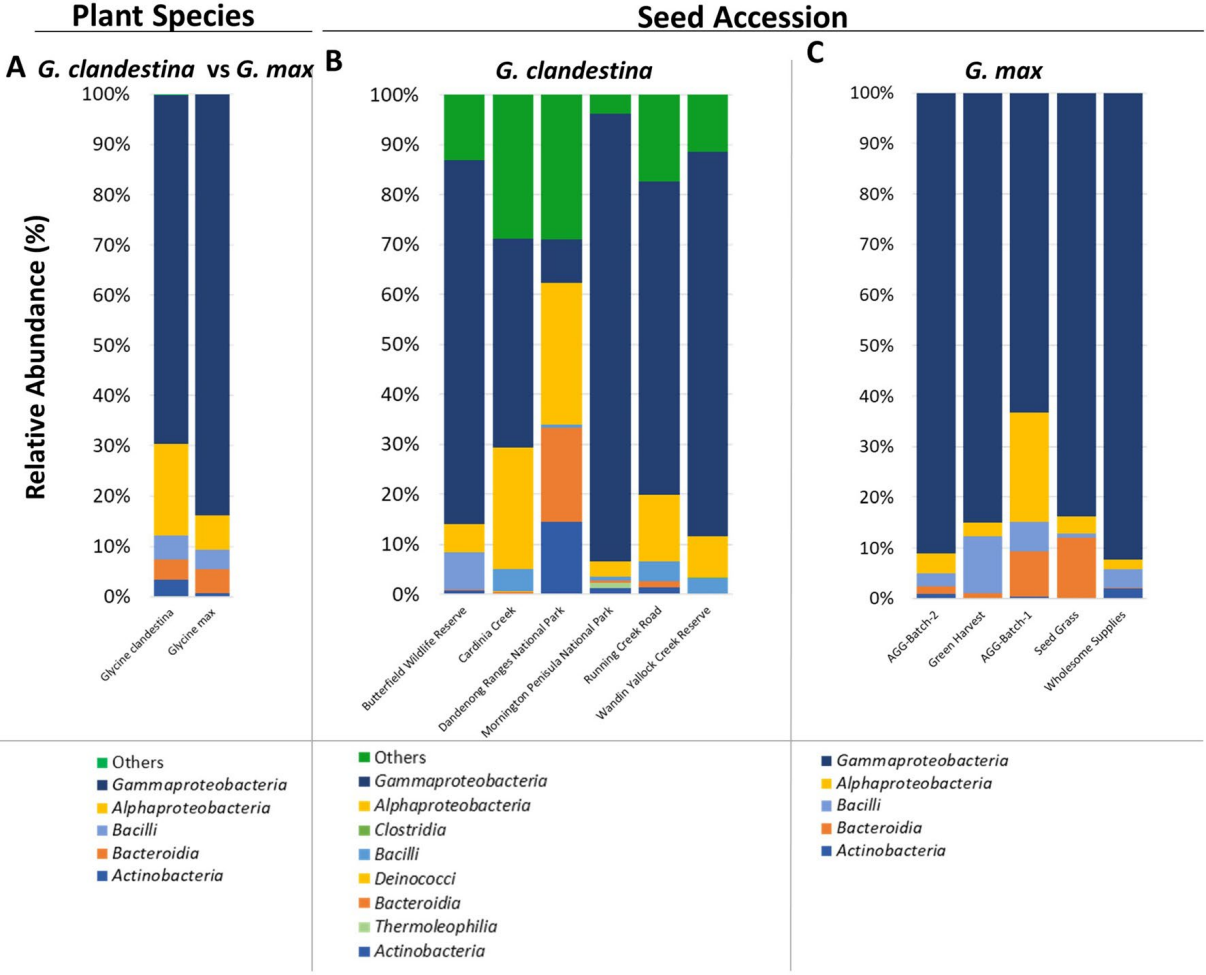
Relative abundance of bacterial communities across all *Glycine* seeds at class level based on plant species (**A**), or seed accession (**B**, **C**). Taxa occurring with less than 0.1% relative abundance are shown as “Others”

**Fig. 3:**
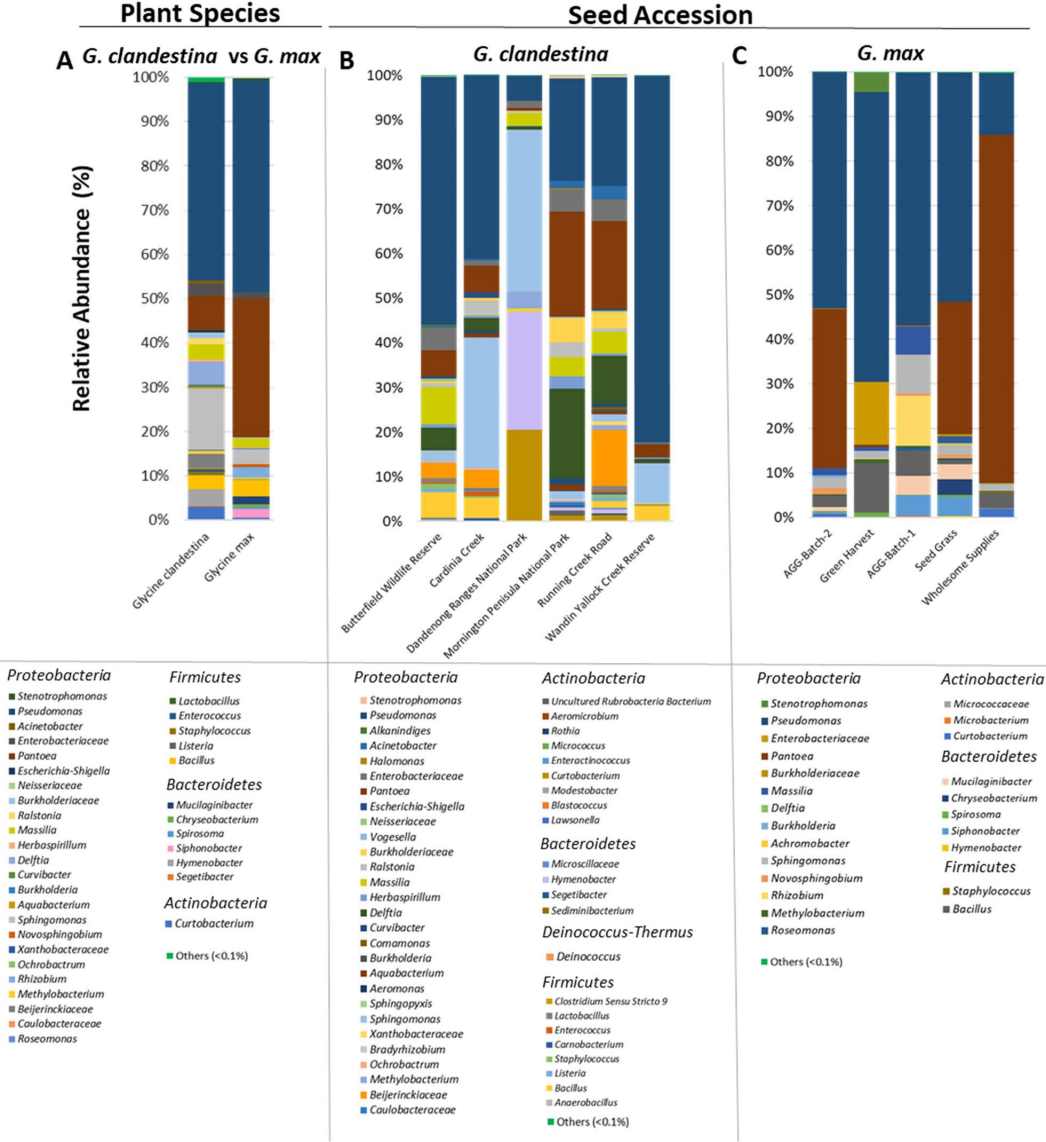
Relative abundance of bacterial communities across all *Glycine* seeds at genus level based on plant species (**A**), or seed accession (**B**, **C**). Taxa occurring with less than 0.1% relative abundance are shown as “Others”

To compare the taxonomic composition in *Glycine* species, ASVs for each accession were first pooled together based on the plant species (Figs. 2A, 3A) and then compared separately for each seed accession (Figs. 2, 3B, C) at the class and genus level, respectively. All taxa represented by < 0.1% of the total number of reads were clustered as “Others”. At the phylum level, *Glycine* seeds were mainly represented by four main phyla (*Proteobacteria*, *Bacteroidetes*, *Firmicutes* and *Actinobacteria*) excluding some low abundant bacterial phyla observed in some seed accessions of *G. clandestina* (Additional file 1: Fig. S4A–C). At the class level, *Glycine* seeds were dominated by *Gammaproteobacteria*. *Gammaproteobacteria* had a 69.4% relative abundance and were represented by 26 genera in *G. clandestina* (Fig. 2A), whereas in *G. max*, the relative abundance was higher (83.7%), but with less diversity, with only 17 genera represented. *Alphaproteobacteria* was the second most dominant class observed, with 18.3% relative abundance in *G. clandestina* and 11 genera, whereas in *G. max*, the relative abundance was lower (7%) with only ten genera. *Bacilli* had a relative abundance of 4.8% and eight genera, followed by *Bacteroidia* with 3.9% relative abundance and six genera in *Glycine clandestina,* compared to *G. max* where the relative abundance was 3.9% and seven genera were members of *Bacilli* and five genera were in *Bacteroidia* (relative abundance 4.7%) (Fig. 2A).

At the genus level, there were 36 genera with a threshold of > 0.1% relative abundance, of which 31 were associated with *G. clandestina* and 27 genera with *G. max* (Fig. 3A, Additional file 1: Table S5). There were 22 genera that were common to both, including *Pseudomonas*, *Pantoea*, *Bacillus* and *Sphingomonas*. There were nine genera unique to *G. clandestina* including *Segetibacter*, *Listeria*, *Beijerinckiaceae*, *Aquabacterium*, *Curvibacter*, *Neisseriaceae*, *Caulobacteraceae*, *Ochrobactrum* and *Xanthobacteraceae*; whilst there were five genera unique to *G. max*, including *Siphonobacter*, *Spirosoma*, *Mucilaginibacter*, *Roseomonas* and *Rhizobium*.

In both cwr and domesticated species, *Pseudomonas* was detected as the most abundant genus (44–48%). Whilst *Sphingomonas* (13.9%), *Pantoea* (8%), *Delftia* (5.12%) and *Hymenobacter* (3.8%) were among the top five ‘highly-abundant’ genera in *G. clandestina.* Whereas *Pantoea* (31.4%), *Bacillus* (3.8%), *Sphingomonas* (3.5%), and *Rhizobium* (2.20%) were the top five dominant genera in *G. max* (Fig. 3A, Additional file 1: Table S5).

The bacterial microbiome composition was then further compared at “Seed Accession” level for each species. At class level in *G. clandestina* seeds, *Gammaproteobacteria* was dominant with a 12.2–93.2% relative abundance and 20 genera, followed by *Alphaproteobaacteria* with a 3.2–40.1% relative abundance and only eight genera (Fig. 2B). Whereas in *G. max* seeds, *Gammaproteobacteria* had a 63.3–92.4% relative abundance and nine genera, followed by *Alphaproteobaacteria* (1.8–21.6%) with only five genera (Fig. 2C). In *G. clandestina*, *Bacilli* had a relative abundance of 0.7–8.7% with seven genera; *Bacteroidia* with 0.5–26.5% relative abundance and four genera and *Actinobacteria* with 0.2–20.5% relative abundance and eight genera. In contrast, with *G. max*, *Bacilli* had a relative abundance of 0.8–11.2% with two genera, followed by *Bacteroidia* (1.4–11.8%) with five genera and *Actinobacteria* with 0.3–2.0% relative abundance and three genera. In addition, some low abundant bacterial classes (< 0.1%) were also detected in some seed accessions of *G. clandestina* grouped as “Others” (Fig. 2B), which were absent in *G. max* (Fig. 2C).

At the genus level, there were 74 genera with a threshold of > 0.1% relative abundance, of which 50 were associated with *G. clandestina* and 24 associated with *G. max* (Fig. 3B, C, Additional file 1: Tables S6, S7). There were only 13 genera shared between all *G. clandestina* seed accessions including *Pseudomonas* (5.6–82.1%), *Sphingomonas* (1.5–36.2%), *Pantoea* (0.5–23.5%), *Delftia* (0.6–20.1%) and *Massilia* (0.1–8.4%). While nine genera were common to *G. max* seed accessions including *Pseudomonas* (13.8–65%), *Pantoea* (0.01–78.1%), *Bacillus* (0.8–11.2%), *Sphingomonas* (1.4–8.8%) and *Massilia* (0.09–6.3%). Notably, the relative abundance of shared genera between cwr and *G. max* were highly variable in the seed accessions of both *Glycine* species (Fig. 3B, C, Additional file 1: Tables S6, S7).

#### Core *Glycine* seed microbiome

**Fig. 4:**
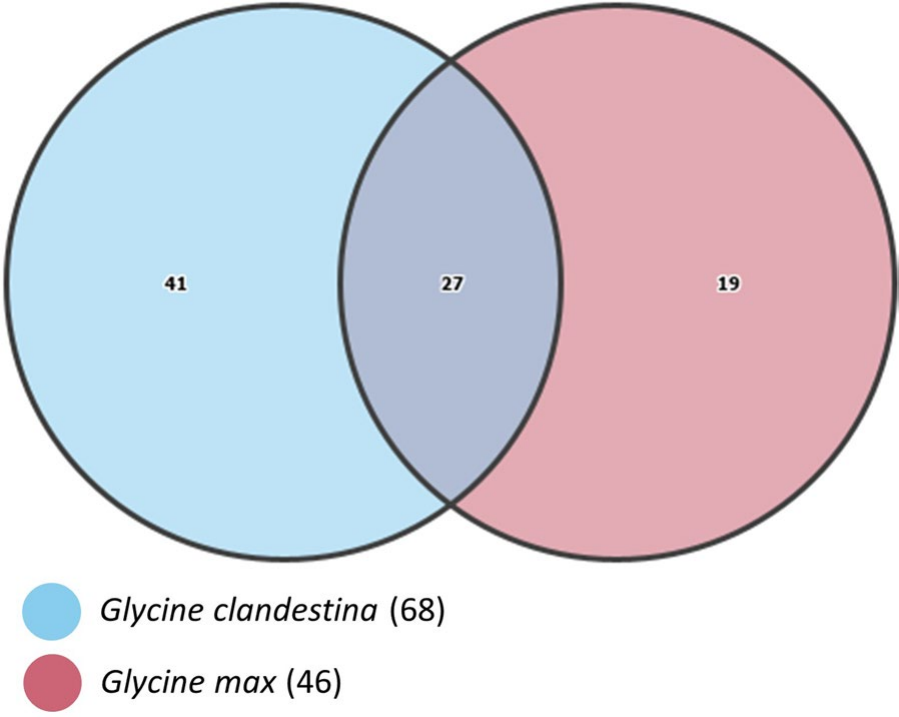
Venn diagrams representing the shared and unique bacterial ASVs associated with *G. clandestina* and *G. max* seed. The numbers in the intersection of the two circles are the shared ASVs, and the remaining numbers are the unique ASVs of both *G. clandestina* and *G. max*

One key aim of this study was to determine the core seed microbiome shared between *G. clandestina* and *G. max*, i.e., the set of bacterial genera found within all *Glycine* seeds independent of “Plant Species” and “Seed Accession”. The core seed microbiome was defined by ASVs (at genus level) present in > 95% of the samples. In this case, 65% of the core *G. max* microbiome had commonality with the cwr, whereas the cwr only had 39% commonality with *G. max*, further indicating the wider microbiome diversity of the cwr *G. clandestina* (Fig. 4).

Overall, the core seed microbiome contained 27 ASVs shared between both *Glycine* species, whilst 41 ASVs were unique to *G. clandestina* and 19 ASVs to *G. max* (Fig. 4). The core seed microbiome represented 93.9% (16,9851 sequences) of sequences from both *Glycine* spp., of which 110,006 (94.2%) sequences were associated with *G. clandestina* and 59,845 (93.5%) with *G. max*. At the class level, *Gammaproteobacteria* was the major component, comprising 44% of the core seed microbiome, followed by *Bacilli* (18.5%), *Alphaproteobacteria* (14.8%), *Actinobacteria* (14.8%) and *Flavobacteria* (7.4%) (Table 2). At the genus level *Pseudomonas* (44.6–47.8%), *Pantoea* (7.9–31.3%), *Sphingomonas* (3.5–13.8%), *Delftia* (0.04–5.1%) and *Bacillus* (3.1–3.8%) were the dominant bacterial genera associated with the core seed microbiome. Of note, the common *G. max* nitrogen-fixing inoculant, *Bradyrhizobium* spp., was identified as part of the core seed microbiome. Furthermore, a common phytopathogenic genus of *G. max*, *Curtobacterium*, was also a member of the core seed microbiome.

**Table 2:** Relative abundance of the core bacterial genera associated with *G. clandestina* and *G. max* seeds

Class	Core bacterial genera	*G. clandestina*	*G. max*
*Gammaproteobacteria*	*Pseudomonas*	**44.604**	**47.884**
	*Pantoea*	**7.951**	**31.394**
	*Delftia*	**5.126**	0.041
	*Massilia*	**3.268**	**2.142**
	*Enterobacteriaceae*	**2.713**	**1.409**
	*Ralstonia*	**1.375**	0.033
	*Burkholderiaceae*	**1.175**	**0.172**
	*Acinetobacter*	**0.682**	0.002
	*Herbaspirillum*	**0.665**	0.013
	*Escherichia-Shigella*	**0.396**	0.008
	*Stenotrophomonas*	**0.183**	**0.420**
	*Burkholderia*	0.052	**0.158**
*Bacilli*	*Bacillus*	**3.136**	**3.808**
	*Staphylococcus*	**0.475**	0.031
	*Lactobacillus*	**0.384**	0.017
	*Enterococcus*	**0.360**	0.006
	*Carnobacterium*	0.070	0.019
*Alphaproteobacteria*	*Sphingomonas*	**13.861**	**3.509**
	*Methylobacterium*	**0.715**	**0.367**
	*Bradyrhizobium*	0.086	0.008
	*Novosphingobium*	0.013	**0.670**
*Actinobacteria*	*Curtobacterium*	**3.110**	**0.558**
	*Micrococcaceae*	0.009	0.039
	*Microbacterium*	0.004	0.072
	*Rhodococcus*	0.004	0.002
*Flavobacteria*	*Hymenobacter*	**3.759**	0.059
	*Chryseobacterium*	0.005	**0.667**

Taxa occurring at > 0.1% are highlighted in bold

### Microbial isolation

#### Microbial identification by MALDI-TOF MS analysis

A total of 117 microbial isolates were obtained from six *G. clandestina* (n = 85, 72% of total) seed accessions and only one *G. max* (n = 32, 28% of total) seed accession. These isolates were then identified at the genus level using MALDI-TOF MS as *Pseudomonas*, *Pantoea*, *Sphingomonas*, *Methylobacterium*, *Curtoacterium*, *Bacillus* and *Rhizobium*, whereas some isolates were not able to be identified by this method. Molecular identification was then performed by selecting 36 isolates representing different clades (both identified and unidentified) from within the MALDI Hierarchical Clustering tree generated (Additional file 2: Fig. S1).

#### Identification of culturable isolates of the *Glycine* seed microbiome

**Fig. 5:**
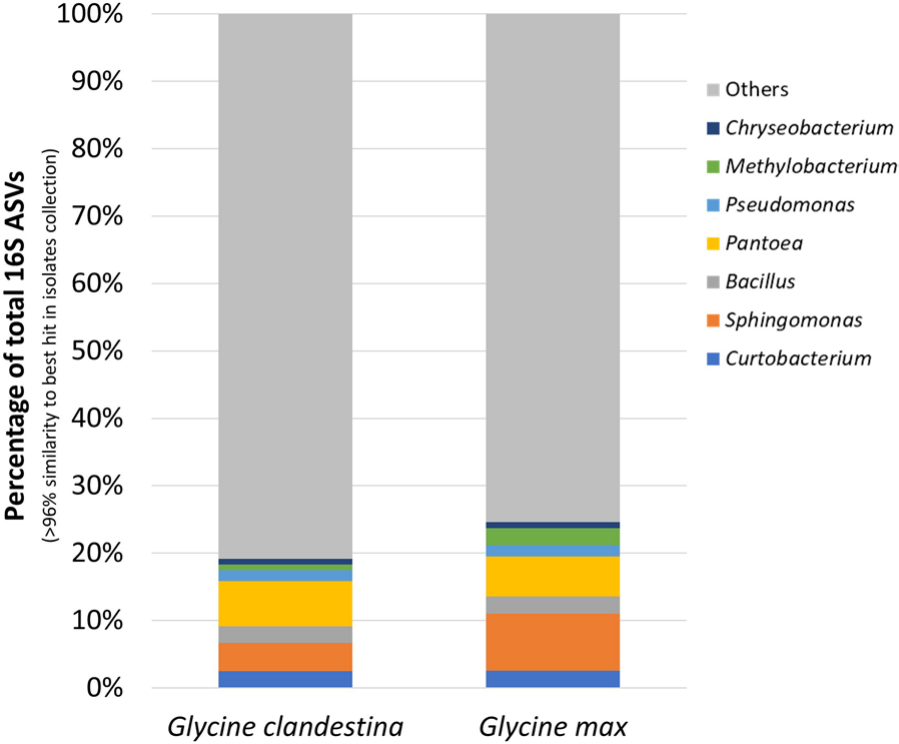
Bar graph showing the percentage of all seed-associated 16S RNA gene ASVs that showed > 96% similarity to at least one Illumina^®^ sequence of the culturable bacteria

After quality filtering and assembly of the 36 isolates using Unicycler (v0.4.8), sequences were taxonomically classified in Kraken2 using an in-house database. Based on Kraken2 classification, 27 of the 36 isolates were linked to eight bacterial genera and nine isolates to two fungal genera. The bacterial isolates were identified as *Pseudomonas*, *Pantoea*, *Sphingomonas*, *Methylobacterium*, *Curtobacterium*, *Streptomyces*, *Bacillus* and *Chryseobacterium*. Similarly, the fungal isolates were identified as *Fusarium* and *Cryptococcus* (Additional file 1: Table S8). When isolated bacterial sequences were mapped to 16S rRNA bacterial gene ASVs from the *Glycine* microbiome, 24.5% of the ASVs associated to *G. max*, and 19.2% ASVs from *G. clandestina*, matched a BLASTn hit against identified bacterial sequences with more than 96% similarity. The majority of the 16S rRNA bacterial gene ASVs in *G. clandestina* (80.8%) and *G. max* (75.5%) were associated with the non-culturable microbiome (Fig. 5).

## Discussion

### Glycine seed microbiota composition

In general, the *Glycine* seed microbiota was occupied by *Gammaproteobacteria, Alphaproteobacteria*, *Bacilli*, *Bacteroidia* and *Actinobacteria*, which is consistent with previous studies concerning domesticated soybean seeds (*Glycine max*) [59], ryegrass (*Lolium perenne*) [60], red sage (*Salvia miltiorrhiza*) [61], rice (*Oryza* sp.) [7, 62], bean (*Phaseolus vulgaris*) [63] and *Brassicaceae* family plants [64], suggesting a commonality between seed microbial communities of *Glycine* and numerous other plants species (Fig. 2A). In particular, we found that *Gammaproteobacteria* was high in both *Glycine* species seeds, which is consistent with previous work about the soybean rhizosphere microbiome [65]. At the genus level, *Pseudomonas* dominated the *Glycine* seed microbiota, as observed in the seed microbiome of other plant species [10, 66]. Berg et al. [24] in their study demonstrated that *Pseudomonas*, *Sphingomonas*, *Tatumella*, *Methylobacterium* and *Pantoea* were the most common bacteria associated with native alpine seeds. This agreed with our findings to some extent, as *Glycine* seeds were mostly populated by *Pseudomonas*, *Sphingomonas* and *Pantoea*, whilst *Methylobacterium* was present in a lower abundance.

### Influence of domestication on *Glycine* seed bacterial diversity

Our study showed that a more diverse seed microbiota was associated with *Glycine* wild relatives compared to the domesticated soybean (Fig. 1A). A higher microbial diversity was also detected within the rhizosphere microbiome of *G. soja* than *G. max* plants [65]. In line with our findings, a recent study showed that seed bacterial diversity was higher in the germinated seedlings of *Triticum dicoccoides* (wild emmer wheat) than the domesticated *Triticum aestivum* (bread wheat) [35]. This implies that selective plant breeding has also led to a shift in composition of the *Glycine* seed microbiome. Several studies have revealed that domestication has resulted in a compositional shift in seed microbial communities of modern cultivars from their wild relatives [7, 28, 67, 68]. The reduced bacterial diversity in *G. max* indicated that, along with the genetic changes in modern cultivars, domestication has also changed the surrounding environmental conditions, thus also altering plant–microbe interactions [69]. This was demonstrated by Longley et al. [70], who showed that different crop management practices impacted the composition of the *G. max* microbiome. In their study they found that the abundance of communities, including the fungal genera *Mortierella*, was high in conventional and organic management systems, while the abundance of genera including the bacteria *Bradyrhizobium* and fungi *Glomeromycotina* was higher under no-till management systems. The use of modern cropping practices, including fertilization, is suggested to alter the microbial composition, functions and interactions due to changes in nutrient availability in surrounding soils [7]. In fact, we found more unique bacterial communities associated with *G. clandestina* than *G. max*. Representatives of the detected unique genera including *Ochrobactrum, Caulobacteraceae, Xanthobacteraceae* and *Beijerinckiaceae* have been identified for their potential to benefit their host plant. For instance, plant beneficial traits such as ability to promote plant health under stress conditions have been documented for *Ochrobactrum* and members of the *Caulobacteraceae* [71, 72], whilst members of *Xanthobacteraceae* and *Beijerinckiaceae* predominantly belonged to the nitrogen-fixing rhizobia [73–75]. Other plant beneficial traits such as biodegradation, bioactivity, quorum sensing and growth promotion have been documented for some *Ochrobactrum* strains isolated from soil, rhizosphere and plant roots [71, 76–78]. This evidence suggests that exposure to a more natural environment (e.g., native soils and climates) could have resulted in the accumulation of more beneficial bacterial communities to potentially support the fitness of native plants, compared to that of modern cultivars of *G. max* [65, 79, 80].

### Key drivers of *Glycine* seed microbiota

The β-diversity analysis showed significant differences in microbiome composition based on factors “Plant Species” and “Seed Accession” (Fig. 1D–F, Additional file 1: Table S4). While microbial communities were significantly more dispersed in *G. clandestina* seed than the *G. max* seed. The influence of host genotype and geographical conditions have also been reported for seed bacterial and fungal profiles of crops seed [60, 81–83]. Although our results indicated that the *Glycine* seed bacterial communities were more influenced by factor “plant species”, as separate clusters formed for seed bacterial communities of *G. clandestina* and *G. max* (Fig. 1D). Previous studies about the surface-sterilized seed of cereals (wheat and barley) [28], maize [84], rice [7, 85] and pumpkin [67] have also demonstrated that unlike rhizosphere/root exudates, the seed bacterial communities are mainly shaped by the factor “plant species” than “geographical locations”. The effect of plant species mainly corresponded to variations in abundance of dominant genera and presence/absence of more unique genera in *G. clandestina*. A recent study by Liu et al. [65] identified that the abundance of *Pseudomonas* and *Pantoea* was limited in the recruited rhizosphere of *G. soja* compared to the rhizosphere of cultivated soybean. This was also in line with our findings, as the relative abundance of *Pseudomonas* and *Pantoea* was higher in *G. max* than *G. clandestina*. Similarly, we found that *Rhizobium* was only associated with *G. max* seed (Additional file 1: Tables S5, S6). On the other hand, *Bradyrhizobium*, a different nitrogen-fixer, was detected in both *G. clandestina* and *G. max* seed, with more abundance in *G. clandestina*, suggesting that some specific strains of rhizobia might be associated with native *Glycine* plants [41] (Additional file 1: Tables S5, S6). This finding was in agreement with a study by Chang and colleagues [86], where they observed an increased abundance of *Bradyrhizobium* strains in the rhizosphere of *G. soja* (wild soybean) than in *G. max*. In an another study, Kim et al. [87] documented that more diverse strains of *Mesorhizobium* were associated with root nodules of wild chickpea (*Cicer reticulatum*) than the cultivated chickpea (*Cicer arietinum*). The influence of host genotype on relative abundance of *Rhizobiales* was also demonstrated in rhizosphere microbiota of tetraploid wheat [88]. This suggested specificity of some plant genotypes towards some microbes was reported by Hanley et al. [89], where the interaction of *Pseudomonas fluorescens* with different accessions of wild *Arabidopsis* was related to the host fitness. The influence of host genotype on the plant microbiome composition has also been documented for the leaf-associated microbiome of *Boechera stricta* (Brassicaceae) by Wagner et al. [90] in a large-scale field experiment.

### Core *Glycine* seed microbiota

Interestingly, despite significant differences between seed microbiota, *G. clandestina* and *G. max* seed did share some core taxa. Core microbial communities are evidence of evolutionary conservation, suggesting that these taxa may have an irreplaceable physiological function for plant seed [29]. Similar observations were documented for the crop seeds such as modern cereals [25] and maize [91], where shared taxa were persistent among wild ancestors and cultivated seeds grown in different geographical locations. The *Glycine* core microbiome was dominated by bacterial ASVs belonging to *Pseudomonas*, *Pantoea* and *Sphingomonas*. Similarly, core bacterial genera such as *Pseudomonas*, *Burkholderia*, *Bacillus*, *Sphingomonas*, *Curtobacterium*, *Methylobacterium*, *Microbacterium*, *Rhizobium* and *Acinetobacter* were also associated with the core seed microbiomes of other plant species [7, 24, 26–29] (Table 2).

All the genera described in the preceding paragraph have been identified for their important roles as endophytes in a variety of plant species. For instance, some strains of *Pseudomonas* (e.g., *Ps. fluorescens*) were identified to fix nitrogen and to act as both a biocontrol agent and a plant growth promoter [92, 93]. Endophytic bacteria isolated from soybean root nodules such as *Pseudomonas*, *Enterobacter*, *Acinetobacter* and *Bacillus* possessed antagonistic properties against *Phytophthora sojae*, have ability to fix nitrogen, produce siderophores and plant hormones (e.g., IAA) [94]. Members of another *Glycine* core genera, *Pantoea*, are known plant pathogens to many agriculturally important plants. Conversely, some strains of *Pantoea* are known for their bioremediation and antimicrobial properties and are now commercially available as biofertilisers (e.g., BlightBan C9-1 and Bloomtime Biological) [95]. On this basis, the role of *Pantoea* with the seed-associated microbiome of *Glycine* still needs further investigation to determine the exact species in this microbiota and thus have a firmer understanding in its role in the *Glycine* microbiome [61]. The other core dominant genus, *Sphingomonas*, has an association with plant root systems and has both bioremediation and plant growth promoting activities [61]. A recent study found that *Sphingomonas* also alleviated reduced plant growth rate and altered the structure of the rhizosphere microbial communities of *Arabidopsis thaliana* under water-deficient conditions [96].

Notably, *Curtobacterium* was also associated with the core *Glycine* microbiome. As reviewed by Chase et al. [97], *Curtobacterium* is globally distributed, prevalent in soil ecosystems and potentially responsible for degradation of organic matter. However, the vertical transmission of the common pathogen *Curtobacterium flaccumfaciens* pv. *flaccumfaciens*, the causal agent of tan spot on soybean leaves, is also well documented [98]. Interestingly, recently genomic analysis of *Curtobacterium* sp. GDI isolated from soybean leaves reported chitinolytic activity, and thus predicted its potential role either as a biocontrol agent, an inducer of plant defence response, a bioremediator, or a simple chitin degrader [99]. Similarly, *Curtobacterium* isolated from the rhizosphere of plants growing in saline conditions was able to alleviate salinity stress, fix nitrogen and to produce plant growth hormones (e.g., ACC, IAA and HCN) [100]. Another important nitrogen-fixing bacterial inoculant of crop seeds, *Bradyrhizobium*, was also associated with the *Glycine* core microbiome. It was demonstrated that the inoculation of commercial soybean with a highly-efficient *Bradyrhizobium* spp. N-fixer provided an alternative nitrogen supply and, as a consequence, dramatically reduced the use of N-fertilizers [101]. Likewise, native *Rhizobium* strains isolated from native cowpea nodules has resulted in enhanced nodulation and plant growth in commercial cowpea when compared to the non-inoculated control [102]. These known attributes suggest that the core microbial taxa can contribute genes essential to confer various benefits to plant health [3].

### Validating the culturability of the *Glycine* microbiome

Another important aspect of our study was to explore the culturable microbiome associated with *Glycine* seeds. Culturing identified *Pseudomonas*, *Pantoea*, *Sphingomonas*, *Methylobacterium*, *Curtobacterium*, *Streptomyces*, *Bacillus* and *Chryseobacterium* (Additional file 1: Table S8). These genera dominated the *Glycine* seed and were also part of the core microbiome (Fig. 3A, Table 1). The culturability of the core seed microbiome has also been reported for rice plants [103] and *Cucurbitaceae* family [104]. The culturable core microbes associated with the *Cucurbitaceae* family, such as *Bacillus*, has been demonstrated to confer various benefits such as growth promotion and nutrient acquisition to their host plant, indicating that the core microbiome is important in the biological and ecological functions of host plants [104]. Notably, two fungal genera including *Fusarium* and *Cryptococcus* were also part of the culturable *Glycine* microbes (Additional file 1: Table S8). Many species of *Fusarium* are known for their pathogenicity in soybean plants [105], although, some of the *Fusarium* strains identified in field grown soybean (roots and seed) were endophytic with a high abundance in roots. It was suggested by Yang et al. [106] that they were either asymptomatic and could become pathogenic under stress conditions or may be true endophytes. Similarly, an endophytic strain of *Cryptococcus* isolated from soybean plant tissues was demonstrated to have cadmium tolerance [107]. The *G. clandestina*-isolated core microbes from this study must be further assessed for their potential benefits to the domesticated *G. max* or to other crop plants, and for their ability to form a stable artificial microbial consortium that can be delivered as a bioprotectant or a biofertilizer [108].

Mapping of the 16S data tag against WGS data showed that the large proportion of the *Glycine* seed microbiome was not present in our culture collection, as only 24.5% of *G. max* and 19.2% of *G. clandestina* ASVs were present (Fig. 5). In contrast to our findings, a higher culturability rate was reported for the seed microbiome of *Lolium perenne* (ryegrass) [66] when using similar growth media (R2A). The low culturability may not be surprising as it was suggested by Sarhan et al. [109] that the auxotrophic and oligo-/prototrophic nature of the microbes could be a reason for their unculturability. For instance, the use of plant-based culture media (e.g., plant-based-sea water media, crude plant juices and plant-only teabags culture media) instead of media of animal or artificial origin (i.e., R2A, LB and NA) has previously demonstrated to increase the culturability of plant microbiomes when tested for barley, cultivated maize, lucerne, cacti, clover, ice plants and desert plants [110–116]

## Conclusion

In conclusion, 16S rRNA profiling of *Glycine* seed microbiome revealed that despite growing under different geographical and climatic conditions, the *Glycine* microbiome composition was found to be primarily influenced by the factor plant species. Moreover, a set of core microbial taxa existed, predominantly dominated by *Gammaproteobacteria,* despite the significantly different microbiome composition between the two species. This outlines the importance of key bacterial genera essential for plant growth, irrespective of the plant genotype and surrounding environment conditions. However, only around 20% of the *Glycine* microbiome was found to be culturable under the culturing conditions of this study. Our findings show that the seed microbiome of native crop species can be used to trace back the microbial communities that might have been lost as a result of domestication as suggested by Berg et al. [34]. We are aware that our dataset for *Glycine* species is comparatively limited. Therefore, our findings cannot be considered conclusive, but they do provide indications of a compositional shift in the seed-associated microbiome due to domestication. This study has shown that a *Glycine* crop wild relative has a more diverse microbiome and provided some of the resources to assess their potential utility in commercial cultivars. Further research characterising the seed microbiome of other native *Glycine* species from vastly different Australian habitats would enhance our understanding about the microbial diversity associated with these crop relatives and the role of natural conditions in accumulation of core seed microbiota.

## Supplementary Information

**Additional file 1. Table S1.** Accession ID, hosts and length of H3-D region of the sequences used for constructing ML tree. **Table S2.** Shannon diversity indices of seed samples grouped by plant species and seed accession. **Table S3.** The Kruskal Wallis pairwise test results calculating significant differences between the bacterial diversity associated with *G. clandestina* and *G. max* seed when samples are grouped based on “Plant Species” and “Seed Accession”. **Table S4.** PERMANOVA and PERMDISP results calculating significant differences in bacterial composition associated with *G. clandestina* and *G. max* when data was grouped based on “Plant Species” and “Seed Accession”. **Table S5.** The relative abundance of bacterial genera associated with both “Plant species”. Taxa occurring at > 0.1% are highlighted in bold. **Table S6.** The relative abundance of bacterial genera associated with different *G. clandestina* seed accessions. Taxa occurring at > 0.1% are highlighted in bold. **Table S7.** The relative abundance of bacterial genera associated with different *G. max* seed accessions. Taxa occurring at > 0.1% are highlighted in bold. **Table S8.** Closest taxonomy ID identified by Kraken2 for the isolated bacterial and fungal sequences with > 96% similarity along with the source of seed accessions. Genome sequences for fungal and bacterial isolates are described under the NCBI BioProjectID PRJNA807720 and PRJNA807698. **Figure S1.** Map highlighting the sites (in yellow) selected for *G. clandestina* seed collection in the Greater Melbourne region, Victoria. **Figure S2.** *G. clandestina* (**A**) and *G. max* (**B**) seedlings at the unfolded cotyledon growth stage. **Figure S3.** Maximum Likelihood Consensus Tree with a bootstrap node support of 70% was inferred from the histone H3-D gene sequences of six Glycine taxa used in this study and the 17 references sequences retrieved from NCBI using MEGA X with 1000 bootstrap replications. The best nucleotide substitution model, the Tamura–Nei model, was used. The ten phylogenetic clades identified within the tree are highlighted in different shades. Sequences reading down (light green, dark green, light blue, yellow, grey, orange, sky blue, violet red and dark red) belong to clades 1 through to 10, respectively. **Figure S4.** The relative abundance of bacterial communities across Glycine seeds at phylum level based on “Plant Species” (**A**), and “Seed Accession” (**B**, **C**).

**Additional file 2. Figure S1.** MALDI-TOF MS spectra generated from a subset of bacteria isolated from *G. clandestina* and *G. max* seeds. The bacterial isolates were identified using an in-house endophyte library and Bruker database. The isolates selected for WGS are labelled next to the representative clade.

## Data Availability

All 16S rRNA amplicon sequence derived from this experiment were submitted into the Short Read Archive of NCBI and can be found under the BioProject accession number PRJNA811248. Assembled genome sequences of all isolates were deposited in the NCBI GenBank and can be found under the BioProject accession number PRJNA807720 for fungal isolates and PRJNA807698 for bacterial isolates.

## Abbreviations

ASVs: Amplicon sequence variants
PERMANOVA: Permutational multivariate ANOVA
bp: Base pair
BLAST: Basic local alignment search tool
CWR: Crop wild relative
DNA: Deoxyribonucleic acid
dsDNA: Double stranded DNA
HCN: Hydrogen cyanide
IAA: Indole-3-acetic acid
LB: Luria–Bertani
K: Potassium
MALDI-TOF: Matrix-assisted laser desorption ionization-time of flight
MS: Mass spectrometry
NCBI: National Center for Biotechnology Information
NGS: Next-generation sequencing
N: Nitrogen
NA: Nutrient agar
PCoA: Principal coordinates analysis
PBS: Phosphate buffered saline
PCR: Polymerase chain reaction
PE: Paired end
PGP: Plant growth-promoting
P: Phosphorous
rRNA: Ribosomal RNA
R2A: Reasoner’s 2A agar
R2B: Reasoner’s 2A broth
SRA: Short read archive
