# Supplementary material for: Australian native Glycine clandestina seed microbiota hosts a more diverse bacterial community than the domesticated soybean Glycine max
Source: Environ Microbiome. 2022 Nov 16;17:56. doi: 10.1186/s40793-022-00452-y (PMC9670509; doi:10.1186/s40793-022-00452-y)
Supplement: Supplementary file 1 — Additional file 1. Table S1. Accession ID, hosts and length of H3-D region of the sequences used for constructing ML tree. Table S2. Shannon diversity indices of seed samples grouped by plant species and seed accession. Table S3. The Kruskal Wallis pairwise test results calculating significant differences between the bacterial diversity associated with G. clandestina and G. max seed when samples are grouped based on “Plant Species” and “Seed Accession”. Table S4. PERMANOVA and PERMDISP results calculating significant differences in bacterial composition associated with G. clandestina and G. max when data was grouped based on “Plant Species” and “Seed Accession”. Table S5. The relative abundance of bacterial genera associated with both “Plant species”. Taxa occurring at > 0.1% are highlighted in bold. Table S6. The relative abundance of bacterial genera associated with different G. clandestina seed accessions. Taxa occurring at > 0.1% are highlighted in bold. Table S7. The relative abundance of bacterial genera associated with different G. max seed accessions. Taxa occurring at > 0.1% are highlighted in bold. Table S8. Closest taxonomy ID identified by Kraken2 for the isolated bacterial and fungal sequences with > 96% similarity along with the source of seed accessions. Genome sequences for fungal and bacterial isolates are described under the NCBI BioProjectID PRJNA807720 and PRJNA807698. Figure S1. Map highlighting the sites (in yellow) selected for G. clandestina seed collection in the Greater Melbourne region, Victoria. Figure S2. G. clandestina (A) and G. max (B) seedlings at the unfolded cotyledon growth stage. Figure S3. Maximum Likelihood Consensus Tree with a bootstrap node support of 70% was inferred from the histone H3-D gene sequences of six Glycine taxa used in this study and the 17 references sequences retrieved from NCBI using MEGA X with 1000 bootstrap replications. The best nucleotide substitution model, the Tamura–Nei model, was used. The ten phylog [file 40793_2022_452_MOESM1_ESM.docx]

**Additional file 1:**

**Australian native *Glycine* *clandestina* seed microbiota host more diverse bacterial communities than the domesticated soybean *Glycine max***

Ankush Chandel^1,2*^, Ross Mann^1^, Jatinder Kaur^1^, Ian Tannenbaum^1^, Sally Norton^3^, Jacqueline Edwards^1,2^, German Spangenberg^1,2^, Tim Sawbridge^1,2^

**Additional file contains:**

Table S1–S8

Figure S1–S4

References

**Table S1** Accession ID, plant sequences (H3-D region) of different Glycine species and length of associated H3-D region used for constructing ML tree.

| **S.no** | **Database ID** | ***Glycine* Host** | **Length** | **References** |
| --- | --- | --- | --- | --- |
| 1 | AF220372.1 | *G.* *canescens* histone H3-D gene, partial cds | 417 bp | [1] |
| 2 | DQ494167.1 | *G.* *canescens* strain G1672 histone H3-D-like gene, partial sequence | 452 bp | [2] |
| 3 | DQ494160.1 | *G. clandestina* strain G1031 histone H3-D-like gene, partial sequence | 451 bp | [2] |
| 4 | DQ494158.1 | *G. clandestina* strain G1225 histone H3-D-like gene, partial sequence | 451 bp | [2] |
| 5 | AF220373.1 | *G.* *hirticaulis* isolate 26 histone H3-D gene, partial cds | 445 bp | [1] |
| 6 | AF493703.1 | *G.* *hirticaulis* clone H3D-hirt3134 histone H3-D gene, exons 2 and 3 and partial cds | 438 bp | [3] |
| 7 | U47404.1 | *G.* *latifolia* histone H3 gene, partial cds, clone H3-DLAF | 468 bp | [4] |
| 8 | AF093435.1 | *G.* *latifolia* cultivar G2545 histone H3-D gene, partial cds | 449 bp | [5] |
| 9 | DQ494170.1 | *G.* *latrobeana* strain G1387 histone H3-D-like gene, partial sequence | 449 bp | [2] |
| 10 | DQ494169.1 | *G.* *latrobeana* strain G1390 histone H3-D-like gene, partial  sequence | 449 bp | [2] |
| 11 | U38424.1 | *G. max* histone H3 gene, partial cds, clone S1 | 690 bp | [6] |
| 12 | U47390.1 | *G.* *microphylla* histone H3 gene, partial cds, clone H3-B2MIC | 454 bp | [4] |
| 13 | U47407.1 | *G.* *microphylla* histone H3 gene, partial cds, clone H3-DMIC | 470 bp | [4] |
| 14 | U47392.1 | *G.* *tabacina* histone H3 gene, partial cds, clone H3-B2TB1138 | 454 bp | [4] |
| 15 | U47409.1 | *G.* *tabacina* histone H3 gene, partial cds, clone H3-DTB1138 | 468 bp | [4] |
| 16 | AF335408.1 | *G.* *tomentella* G3119 histone H3 (H3-D) gene, partial cds | 453 bp | [7] |
| 17 | AF335406.1 | *G.* *tomentella* G2576 histone H3 (H3-D) gene, partial cds | 453 bp | [7] |

**Table S2** Shannon diversity indices of seed samples grouped by plant species and seed accession.

|  | | | **Shannon Diversity Index** |
| --- | --- | --- | --- |
| **Plant Species** | | *G. clandestina* | 2.42 |
|  |  | *G. max* | 1.17 |
| **Seed Accessions** | ***G. clandestina*** | Butterfield Wildlife Reserve | 3.17 |
|  |  | Cardinia Creek | 1.43 |
|  |  | Dandenong Ranges National Park | 2.97 |
|  |  | Mornington Peninsula National Park | 3.09 |
|  |  | Running Creek Road | 2.73 |
|  |  | Wandin Yallock Creek Reserve | 2.11 |
|  | ***G. max*** | AGG-Batch-1 | 1.13 |
|  |  | Green Harvest | 1.58 |
|  |  | AGG-Batch-2 | 1.02 |
|  |  | Seed Grass | 1.50 |
|  |  | Wholesome Supplies | 0.98 |

**Table S3** The Kruskal Wallis pairwise test results calculating significant differences between the bacterial diversity associated with G. clandestina and G. max seed when samples are grouped based on “Plant Species” and “Seed Accession”.

|  | | **Group 1** | **Group 2** | ***p-*values** |
| --- | --- | --- | --- | --- |
| **Plant Species** | | *G. clandestina* | *G. max* | **0.000042** |
| **Seed Accessions** | ***G. clandestina*** | Butterfield Wildlife Reserve | Cardinia Creek | 0.111479 |
|  |  | Butterfield Wildlife Reserve | Dandenong Ranges National Park | 0.336739 |
|  |  | Butterfield Wildlife Reserve | Mornington Peninsula National Park | 0.111479 |
|  |  | Butterfield Wildlife Reserve | Running Creek Road | 0.092589 |
|  |  | Butterfield Wildlife Reserve | Wandin Yallock Creek Reserve | **0.000092** |
|  |  | Cardinia Creek | Dandenong Ranges National Park | 0.617617 |
|  |  | Cardinia Creek | Mornington Peninsula National Park | **0.041187** |
|  |  | Cardinia Creek | Running Creek Road | **0.019917** |
|  |  | Cardinia Creek | Wandin Yallock Creek Reserve | **0.000760** |
|  |  | Dandenong Ranges National Park | Mornington Peninsula National Park | **0.041326** |
|  |  | Dandenong Ranges National Park | Running Creek Road | **0.038341** |
|  |  | Dandenong Ranges National Park | Wandin Yallock Creek Reserve | **0.002230** |
|  |  | Mornington Peninsula National Park | Running Creek Road | 0.526534 |
|  |  | Mornington Peninsula National Park | Wandin Yallock Creek Reserve | **0.000760** |
|  |  | Running Creek Road | Wandin Yallock Creek Reserve | **0.000343** |
|  | ***G. max*** | AGG-Batch-2 | Green Harvest | 0.924500 |
|  |  | AGG-Batch-2 | AGG-Batch-2 | 0.961261 |
|  |  | AGG-Batch-2 | Seed Grass | 0.924500 |
|  |  | AGG-Batch-2 | Wholesome Supplies | 0.961261 |
|  |  | Green Harvest | AGG-Batch-2 | 0.961261 |
|  |  | Green Harvest | Seed Grass | 0.961261 |
|  |  | Green Harvest | Wholesome Supplies | 0.924500 |
|  |  | AGG-Batch-1 | Seed Grass | 0.924500 |
|  |  | AGG-Batch-1 | Wholesome Supplies | 1.000000 |
|  |  | Seed Grass | Wholesome Supplies | 0.491408 |

Note: Significant differences indicated in **bold** (*p<*0.05).

**Table S4** PERMANOVA and PERMDISP results calculating significant differences in bacterial composition associated with *G. clandestina* and *G. max* when data was grouped based on “Plant Species” and “Seed Accession”.

|  | | | | **PERMANOVA** | **PERMDISP** |
| --- | --- | --- | --- | --- | --- |
|  |  |  |  | **Unweighed** | **Unweighed** |
|  | | **Group 1** | **Group 2** | ***p*-value** | ***p*-value** |
| **Plant Species** | | *G. clandestina* | *G. max* | **0.001** | **0.005** |
| **Seed Accessions** | ***G. clandestina*** | Butterfield Wildlife Reserve | Cardinia Creek | **0.001** | 0.575 |
|  |  | Butterfield Wildlife Reserve | Dandenong Ranges National Park | **0.001** | **0.005** |
|  |  | Butterfield Wildlife Reserve | Mornington Peninsula National Park | **0.001** | 0.858 |
|  |  | Butterfield Wildlife Reserve | Running Creek Road | **0.004** | 0.858 |
|  |  | Butterfield Wildlife Reserve | Wandin Yallock Creek Reserve | **0.001** | **0.036** |
|  |  | Cardinia Creek | Dandenong Ranges National Park | **0.001** | **0.005** |
|  |  | Cardinia Creek | Mornington Peninsula National Park | **0.001** | 0.631 |
|  |  | Cardinia Creek | Running Creek Road | **0.001** | 0.639 |
|  |  | Cardinia Creek | Wandin Yallock Creek Reserve | **0.001** | **0.015** |
|  |  | Dandenong Ranges National Park | Mornington Peninsula National Park | **0.001** | **0.008** |
|  |  | Dandenong Ranges National Park | Running Creek Road | **0.001** | **0.036** |
|  |  | Dandenong Ranges National Park | Wandin Yallock Creek Reserve | **0.001** | **0.005** |
|  |  | Mornington Peninsula National Park | Running Creek Road | **0.015** | 0.939 |
|  |  | Mornington Peninsula National Park | Wandin Yallock Creek Reserve | **0.001** | 0.171 |
|  |  | Running Creek Road | Wandin Yallock Creek Reserve | **0.001** | 0.248 |
|  | ***G. max*** | AGG-Batch-2 | Green Harvest | **0.023** | 0.388 |
|  |  | AGG-Batch-2 | AGG-Batch-2 | **0.003** | 0.420 |
|  |  | AGG-Batch-2 | Seed Grass | **0.003** | 0.367 |
|  |  | AGG-Batch-2 | Wholesome Supplies | **0.003** | 0.420 |
|  |  | Green Harvest | AGG-Batch-2 | **0.003** | 0.150 |
|  |  | Green Harvest | Seed Grass | **0.003** | 0.203 |
|  |  | Green Harvest | Wholesome Supplies | **0.016** | 0.203 |
|  |  | AGG-Batch-1 | Seed Grass | **0.003** | 0.420 |
|  |  | AGG-Batch-1 | Wholesome Supplies | **0.003** | 0.692 |
|  |  | Seed Grass | Wholesome Supplies | **0.003** | 0.420 |

Note: Significant differences indicated in **bold** (*p<*0.05).

**Table S5** The relative abundance of bacterial genera associated with both “Plant species”. Taxa occurring at >0.1% are highlighted in bold.

| **Taxonomic level** | | | **Relative abundance in both "Plant Species" (%)** | |
| --- | --- | --- | --- | --- |
|  |  |  |  |  |
| **Phylum** | **Family** | **Genus** | ***G. clandestina*** | ***G. max*** |
|  |  |  |  |  |
| *Acidobacteria* | *Acidobacteria* | Unclassified | 0.001 | 0.000 |
| *Actinobacteria* | *Corynebacteriaceae* | *Lawsonella* | 0.058 | 0.000 |
| *Actinobacteria* | *Nocardiaceae* | *Rhodococcus* | 0.004 | 0.002 |
| *Actinobacteria* | *Geodermatophilaceae* | *Blastococcus* | 0.021 | 0.000 |
| *Actinobacteria* | *Geodermatophilaceae* | *Modestobacter* | 0.033 | 0.000 |
| *Actinobacteria* | *Cellulomonadaceae* | *Cellulomonas* | 0.000 | 0.002 |
| *Actinobacteria* | *Dermabacteraceae* | *Brachybacterium* | 0.006 | 0.000 |
| *Actinobacteria* | *Microbacteriaceae* | *Curtobacterium* | **3.110** | **0.558** |
| *Actinobacteria* | *Microbacteriaceae* | *Leifsonia* | 0.000 | 0.003 |
| *Actinobacteria* | *Microbacteriaceae* | *Microbacterium* | 0.004 | 0.072 |
| *Actinobacteria* | *Micrococcaceae* | *Enteractinococcus* | 0.060 | 0.000 |
| *Actinobacteria* | *Micrococcaceae* | *Micrococcus* | 0.018 | 0.000 |
| *Actinobacteria* | *Micrococcaceae* | *Rothia* | 0.019 | 0.000 |
| *Actinobacteria* | *Micrococcaceae* | Unclassified | 0.009 | 0.039 |
| *Actinobacteria* | *Nocardioidaceae* | *Aeromicrobium* | 0.042 | 0.000 |
| *Actinobacteria* | *Propionibacteriaceae* | *Cutibacterium* | 0.017 | 0.000 |
| *Actinobacteria* | *67-14* | *Uncultured Rubrobacteria bacterium* | 0.099 | 0.000 |
| *Bacteroidetes* | *Prevotellaceae* | *Prevotella 9* | 0.003 | 0.000 |
| *Bacteroidetes* | *Chitinophagaceae* | *Sediminibacterium* | 0.010 | 0.000 |
| *Bacteroidetes* | *Chitinophagaceae* | *Segetibacter* | **0.126** | 0.000 |
| *Bacteroidetes* | *Hymenobacteraceae* | *Hymenobacter* | **3.759** | 0.059 |
| *Bacteroidetes* | *Microscillaceae* | *Siphonobacter* | 0.000 | **1.913** |
| *Bacteroidetes* | *Microscillaceae* | Uncultured | 0.039 | 0.000 |
| *Bacteroidetes* | *Spirosomaceae* | *Spirosoma* | 0.000 | **0.320** |
| *Bacteroidetes* | *Weeksellaceae* | *Chryseobacterium* | 0.005 | **0.667** |
| *Bacteroidetes* | *Sphingobacteriaceae* | *Mucilaginibacter* | 0.000 | **1.773** |
| *Bacteroidetes* | *Sphingobacteriaceae* | *Pedobacter* | 0.000 | 0.002 |
| *Bacteroidetes* | *env.OPS 17* | Unclassified | 0.000 | 0.002 |
| *Cyanobacteria* | *Uncultured bacterium* | *Uncultured bacterium* | 0.002 | 0.000 |
| *Deinococcus-Thermus* | *Deinococcaceae* | *Deinococcus* | 0.037 | 0.000 |
| *Firmicutes* | *Bacillaceae* | *Anaerobacillus* | 0.046 | 0.000 |
| *Firmicutes* | *Bacillaceae* | *Bacillus* | **3.136** | **3.808** |
| *Firmicutes* | *Listeriaceae* | *Listeria* | **0.296** | 0.000 |
| *Firmicutes* | *Staphylococcaceae* | *Staphylococcus* | **0.475** | 0.031 |
| *Firmicutes* | *Carnobacteriaceae* | *Carnobacterium* | 0.070 | 0.019 |
| *Firmicutes* | *Enterococcaceae* | *Enterococcus* | **0.360** | 0.006 |
| *Firmicutes* | *Lactobacillaceae* | *Lactobacillus* | **0.384** | 0.017 |
| *Firmicutes* | *Clostridiaceae 1* | *Clostridium sensu stricto 9* | 0.042 | 0.000 |
| *Proteobacteria* | *Acetobacteraceae* | *Acidiphilium* | 0.015 | 0.000 |
| *Proteobacteria* | *Acetobacteraceae* | *Roseomonas* | 0.000 | **0.192** |
| *Proteobacteria* | *Caulobacteraceae* | *Asticcacaulis* | 0.000 | 0.003 |
| *Proteobacteria* | *Caulobacteraceae* | Uncultured | **0.116** | 0.000 |
| *Proteobacteria* | *Micropepsaceae* | Uncultured | 0.001 | 0.000 |
| *Proteobacteria* | *Beijerinckiaceae* | Unclassified | **3.128** | 0.000 |
| *Proteobacteria* | *Beijerinckiaceae* | *Methylobacterium* | **0.715** | **0.367** |
| *Proteobacteria* | *Beijerinckiaceae* | *Microvirga* | 0.000 | 0.008 |
| *Proteobacteria* | *Rhizobiaceae* | *Allorhizobium-Neorhizobium-Pararhizobium-Rhizobium* | 0.000 | **2.205** |
| *Proteobacteria* | *Rhizobiaceae* | *Aureimonas* | 0.000 | 0.002 |
| *Proteobacteria* | *Rhizobiaceae* | *Ochrobactrum* | **0.164** | 0.000 |
| *Proteobacteria* | *Rhizobiaceae* | Unclassified | 0.000 | 0.005 |
| *Proteobacteria* | *Xanthobacteraceae* | *Bradyrhizobium* | 0.086 | 0.008 |
| *Proteobacteria* | *Xanthobacteraceae* | Unclassified | **0.115** | 0.000 |
| *Proteobacteria* | *Sneathiellaceae* | *Ferrovibrio* | 0.000 | 0.002 |
| *Proteobacteria* | *Sphingomonadaceae* | *Novosphingobium* | 0.013 | **0.670** |
| *Proteobacteria* | *Sphingomonadaceae* | *Sphingomonas* | **13.861** | **3.509** |
| *Proteobacteria* | *Sphingomonadaceae* | *Sphingopyxis* | 0.049 | 0.000 |
| *Proteobacteria* | *Aeromonadaceae* | *Aeromonas* | 0.015 | 0.000 |
| *Proteobacteria* | *Burkholderiaceae* | *Achromobacter* | 0.000 | 0.055 |
| *Proteobacteria* | *Burkholderiaceae* | *Aquabacterium* | **0.386** | 0.000 |
| *Proteobacteria* | *Burkholderiaceae* | *Burkholderia-Caballeronia-Paraburkholderia* | 0.052 | **0.158** |
| *Proteobacteria* | *Burkholderiaceae* | *Castellaniella* | 0.000 | 0.003 |
| *Proteobacteria* | *Burkholderiaceae* | *Comamonas* | 0.091 | 0.000 |
| *Proteobacteria* | *Burkholderiaceae* | *Curvibacter* | **0.403** | 0.000 |
| *Proteobacteria* | *Burkholderiaceae* | *Delftia* | **5.126** | 0.041 |
| *Proteobacteria* | *Burkholderiaceae* | *Duganella* | 0.001 | 0.000 |
| *Proteobacteria* | *Burkholderiaceae* | *Herbaspirillum* | **0.665** | 0.013 |
| *Proteobacteria* | *Burkholderiaceae* | *Hydrogenophaga* | 0.005 | 0.000 |
| *Proteobacteria* | *Burkholderiaceae* | *Massilia* | **3.268** | **2.142** |
| *Proteobacteria* | *Burkholderiaceae* | *Ralstonia* | **1.375** | 0.033 |
| *Proteobacteria* | *Burkholderiaceae* | Unclassified | **1.175** | **0.172** |
| *Proteobacteria* | *Chromobacteriaceae* | *Vogesella* | 0.022 | 0.000 |
| *Proteobacteria* | *Methylophilaceae* | *Methylophilus* | 0.000 | 0.002 |
| *Proteobacteria* | *Neisseriaceae* | *Neisseria* | 0.003 | 0.000 |
| *Proteobacteria* | *Neisseriaceae* | Uncultured | **0.207** | 0.000 |
| *Proteobacteria* | *Enterobacteriaceae* | *Escherichia-Shigella* | **0.396** | 0.008 |
| *Proteobacteria* | *Enterobacteriaceae* | *Pantoea* | **7.951** | **31.394** |
| *Proteobacteria* | *Enterobacteriaceae* | Unclassified | **2.713** | **1.409** |
| *Proteobacteria* | *Unknown Family* | *Acidibacter* | 0.000 | 0.002 |
| *Proteobacteria* | *Halomonadaceae* | *Halomonas* | 0.033 | 0.000 |
| *Proteobacteria* | *Pasteurellaceae* | *Haemophilus* | 0.004 | 0.000 |
| *Proteobacteria* | *Moraxellaceae* | *Acinetobacter* | **0.682** | 0.002 |
| *Proteobacteria* | *Moraxellaceae* | *Alkanindiges* | 0.079 | 0.000 |
| *Proteobacteria* | *Pseudomonadaceae* | *Pseudomonas* | **44.604** | **47.884** |
| *Proteobacteria* | *Steroidobacteraceae* | *Steroidobacter* | 0.001 | 0.000 |
| *Proteobacteria* | *Xanthomonadaceae* | *Stenotrophomonas* | **0.183** | **0.420** |
| *Proteobacteria* | *Xanthomonadaceae* | *Thermomonas* | 0.000 | 0.002 |
| *Proteobacteria* | *Xanthomonadaceae* | Unclassified | 0.001 | 0.000 |
| Bacteria | Bacteria | Unclassified | 0.004 | 0.000 |

**Table S6** The relative abundance of bacterial genera associated with different G. clandestina seed accessions. Taxa occurring at >0.1% are highlighted in bold.

| **Taxonomic level** | | **Relative abundance in *G. clandestina* "Seed Accessions" (%)** | | | | | | |
| --- | --- | --- | --- | --- | --- | --- | --- | --- |
|  |  |  |  |  |  |  |  |  |
| **Phylum** | **Class** | **Genus** | **Butterfield Wildlife Reserve** | **Cardinia Creek** | **Dandenong Ranges National Park** | **Mornington Peninsula National Park** | **Running Creek Road** | **Wandin Yallock Creek Reserve** |
| *Acidobacteria* | *Subgroup 6* | *Unclassified* | 0.000 | 0.004 | 0.000 | 0.000 | 0.000 | 0.000 |
| *Actinobacteria* | *Actinobacteria* | *Lawsonella* | **0.223** | 0.000 | 0.000 | 0.000 | 0.000 | 0.000 |
| *Actinobacteria* | *Actinobacteria* | *Rhodococcus* | 0.043 | 0.000 | 0.000 | 0.000 | 0.000 | 0.000 |
| *Actinobacteria* | *Actinobacteria* | *Blastococcus* | 0.000 | 0.000 | 0.000 | 0.000 | **0.139** | 0.000 |
| *Actinobacteria* | *Actinobacteria* | *Modestobacter* | **0.164** | 0.000 | 0.000 | 0.000 | 0.000 | 0.000 |
| *Actinobacteria* | *Actinobacteria* | *Brachybacterium* | 0.027 | 0.000 | 0.000 | 0.000 | 0.000 | 0.000 |
| *Actinobacteria* | *Actinobacteria* | *Curtobacterium* | 0.074 | **0.108** | **20.519** | **1.286** | **1.160** | **0.219** |
| *Actinobacteria* | *Actinobacteria* | *Microbacterium* | 0.000 | 0.013 | 0.000 | 0.000 | 0.000 | 0.000 |
| *Actinobacteria* | *Actinobacteria* | *Enteractinococcus* | **0.227** | 0.000 | 0.000 | 0.000 | 0.000 | 0.000 |
| *Actinobacteria* | *Actinobacteria* | *Micrococcus* | 0.000 | 0.000 | 0.000 | 0.000 | **0.167** | 0.000 |
| *Actinobacteria* | *Actinobacteria* | *Rothia* | 0.000 | 0.000 | 0.000 | 0.000 | **0.208** | 0.000 |
| *Actinobacteria* | *Actinobacteria* | *Unclassified* | 0.027 | 0.000 | 0.000 | 0.000 | 0.000 | 0.008 |
| *Actinobacteria* | *Actinobacteria* | *Aeromicrobium* | **0.129** | 0.000 | 0.000 | 0.000 | 0.000 | 0.000 |
| *Actinobacteria* | *Actinobacteria* | *Cutibacterium* | 0.039 | 0.004 | 0.000 | 0.045 | 0.000 | 0.000 |
| *Actinobacteria* | *Thermoleophilia* | *Uncultured Rubrobacteria bacterium* | 0.000 | 0.000 | 0.000 | **1.071** | 0.000 | 0.000 |
| *Bacteroidetes* | *Bacteroidia* | *Prevotella 9* | 0.023 | 0.000 | 0.000 | 0.000 | 0.000 | 0.000 |
| *Bacteroidetes* | *Bacteroidia* | *Sediminibacterium* | 0.000 | 0.000 | 0.000 | 0.000 | **0.132** | 0.000 |
| *Bacteroidetes* | *Bacteroidia* | *Segetibacter* | 0.000 | **0.592** | 0.000 | 0.000 | 0.000 | 0.000 |
| *Bacteroidetes* | *Bacteroidia* | *Hymenobacter* | 0.000 | 0.000 | **26.450** | **0.527** | **0.826** | 0.000 |
| *Bacteroidetes* | *Bacteroidia* | *Uncultured bacterium* | 0.000 | 0.000 | 0.000 | 0.000 | **0.403** | 0.000 |
| *Bacteroidetes* | *Bacteroidia* | *Chryseobacterium* | 0.000 | 0.000 | 0.019 | 0.000 | 0.028 | 0.000 |
| *Chloroflexi* | *Chloroflexia* | *Paraburkholderia tropica* | 0.000 | 0.000 | 0.000 | 0.000 | 0.000 | 0.008 |
| *Cyanobacteria* | *Melainabacteria* | *Uncultured bacterium* | 0.000 | 0.000 | 0.000 | 0.000 | 0.000 | 0.008 |
| *Deinococcus-Thermus* | *Deinococci* | *Deinococcus* | 0.000 | **0.150** | 0.000 | 0.000 | 0.000 | 0.000 |
| *Firmicutes* | *Bacilli* | *Anaerobacillus* | 0.000 | 0.063 | 0.000 | **0.295** | 0.000 | 0.000 |
| *Firmicutes* | *Bacilli* | *Bacillus* | **5.680** | **4.333** | **0.706** | 0.027 | **1.444** | **3.152** |
| *Firmicutes* | *Bacilli* | *Listeria* | **0.777** | 0.075 | 0.000 | 0.000 | **0.819** | 0.023 |
| *Firmicutes* | *Bacilli* | *Staphylococcus* | **1.184** | **0.308** | 0.019 | 0.000 | **0.792** | **0.172** |
| *Firmicutes* | *Bacilli* | *Carnobacterium* | 0.000 | 0.000 | 0.000 | **0.375** | **0.278** | 0.000 |
| *Firmicutes* | *Bacilli* | *Enterococcus* | **0.340** | **1.004** | 0.013 | 0.000 | **0.521** | 0.008 |
| *Firmicutes* | *Bacilli* | *Lactobacillus* | **0.797** | **0.471** | 0.000 | 0.000 | **1.000** | 0.012 |
| *Firmicutes* | *Clostridia* | *Clostridium sensu stricto 9* | 0.000 | 0.000 | 0.000 | 0.000 | 0.000 | **0.223** |
| *Proteobacteria* | *Alphaproteobacteria* | *Acidiphilium* | 0.000 | 0.000 | 0.000 | 0.000 | 0.000 | 0.066 |
| *Proteobacteria* | *Alphaproteobacteria* | *Uncultured bacterium* | 0.000 | **0.283** | 0.000 | **0.696** | 0.000 | 0.000 |
| *Proteobacteria* | *Alphaproteobacteria* | *Uncultured bacterium* | 0.000 | 0.000 | 0.013 | 0.000 | 0.000 | 0.000 |
| *Proteobacteria* | *Alphaproteobacteria* | *1174-901-12* | **3.445** | **4.163** | 0.000 | 0.000 | **12.597** | 0.039 |
| *Proteobacteria* | *Alphaproteobacteria* | *Methylobacterium* | **0.152** | 0.000 | **3.750** | **0.268** | **1.069** | 0.031 |
| *Proteobacteria* | *Alphaproteobacteria* | *Ochrobactrum* | **0.277** | **0.371** | 0.000 | 0.071 | 0.090 | 0.059 |
| *Proteobacteria* | *Alphaproteobacteria* | *Bradyrhizobium* | 0.066 | 0.000 | 0.038 | **0.527** | 0.000 | 0.082 |
| *Proteobacteria* | *Alphaproteobacteria* | Unclassified | 0.023 | 0.017 | 0.000 | 0.000 | **0.750** | 0.098 |
| *Proteobacteria* | *Alphaproteobacteria* | *Novosphingobium* | 0.035 | 0.000 | 0.044 | 0.000 | 0.000 | 0.008 |
| *Proteobacteria* | *Alphaproteobacteria* | *Sphingomonas* | **2.145** | **29.258** | **36.225** | **1.634** | **1.597** | **8.965** |
| *Proteobacteria* | *Alphaproteobacteria* | *Sphingopyxis* | **0.246** | 0.000 | 0.000 | 0.000 | 0.000 | 0.000 |
| *Proteobacteria* | *Gammaproteobacteria* | *Aeromonas* | 0.000 | 0.000 | 0.000 | **0.179** | 0.000 | 0.000 |
| *Proteobacteria* | *Gammaproteobacteria* | *Aquabacterium* | 0.043 | **0.879** | 0.075 | **1.330** | **0.479** | **0.125** |
| *Proteobacteria* | *Gammaproteobacteria* | *Burkholderia-Caballeronia-Paraburkholderia* | 0.000 | 0.000 | 0.000 | 0.000 | **0.472** | 0.000 |
| *Proteobacteria* | *Gammaproteobacteria* | *Comamonas* | 0.000 | 0.000 | 0.000 | 0.000 | **0.736** | 0.000 |
| *Proteobacteria* | *Gammaproteobacteria* | *Curvibacter* | **0.219** | **0.688** | 0.000 | **1.402** | **0.708** | 0.047 |
| *Proteobacteria* | *Gammaproteobacteria* | *Delftia* | **4.824** | **2.842** | **0.681** | **20.116** | **10.667** | **0.742** |
| *Proteobacteria* | *Gammaproteobacteria* | *Duganella* | 0.000 | 0.000 | 0.000 | 0.000 | 0.000 | 0.004 |
| *Proteobacteria* | *Gammaproteobacteria* | *Herbaspirillum* | **0.680** | **0.508** | **0.169** | **2.714** | **0.590** | **0.160** |
| *Proteobacteria* | *Gammaproteobacteria* | *Hydrogenophaga* | 0.000 | 0.000 | 0.000 | 0.063 | 0.000 | 0.000 |
| *Proteobacteria* | *Gammaproteobacteria* | *Massilia* | **8.449** | **0.108** | **2.806** | **4.277** | **4.868** | 0.027 |
| *Proteobacteria* | *Gammaproteobacteria* | *Ralstonia* | **0.809** | **3.275** | **0.444** | **3.250** | **0.736** | 0.090 |
| *Proteobacteria* | *Gammaproteobacteria* | Unclassified | **0.336** | **0.617** | **0.156** | **5.589** | **3.549** | 0.055 |
| *Proteobacteria* | *Gammaproteobacteria* | *Vogesella* | 0.000 | 0.000 | 0.000 | **0.205** | 0.000 | 0.000 |
| *Proteobacteria* | *Gammaproteobacteria* | *Neisseria* | 0.000 | 0.013 | 0.000 | 0.000 | 0.000 | 0.000 |
| *Proteobacteria* | *Gammaproteobacteria* | *Uncultured bacterium* | **0.730** | 0.000 | 0.000 | 0.000 | **0.389** | 0.000 |
| *Proteobacteria* | *Gammaproteobacteria* | *Escherichia-Shigella* | **0.488** | **1.183** | 0.000 | 0.000 | **0.340** | 0.008 |
| *Proteobacteria* | *Gammaproteobacteria* | *Pantoea* | **5.762** | **6.008** | **0.575** | **23.527** | **19.833** | **2.898** |
| *Proteobacteria* | *Gammaproteobacteria* | Unclassified | **5.180** | **1.113** | **1.644** | **5.054** | **4.799** | **0.348** |
| *Proteobacteria* | *Gammaproteobacteria* | *Halomonas* | 0.000 | 0.058 | 0.000 | **0.188** | 0.000 | 0.000 |
| *Proteobacteria* | *Gammaproteobacteria* | *Haemophilus* | 0.000 | 0.000 | 0.000 | 0.000 | 0.000 | 0.012 |
| *Proteobacteria* | *Gammaproteobacteria* | *Acinetobacter* | **0.215** | **0.350** | 0.013 | **1.634** | **2.986** | **0.203** |
| *Proteobacteria* | *Gammaproteobacteria* | *Alkanindiges* | **0.355** | 0.000 | 0.000 | 0.000 | 0.000 | 0.000 |
| *Proteobacteria* | *Gammaproteobacteria* | *Pseudomonas* | **55.598** | **41.108** | **5.613** | **22.964** | **24.319** | **82.098** |
| *Proteobacteria* | *Gammaproteobacteria* | *Stenotrophomonas* | **0.168** | 0.000 | 0.031 | **0.688** | **0.507** | 0.000 |
| *Proteobacteria* | *Gammaproteobacteria* | Unclassified | 0.000 | 0.000 | 0.000 | 0.000 | 0.000 | 0.004 |
| Unclassified | Unclassified | Unclassified | 0.000 | 0.033 | 0.000 | 0.000 | 0.000 | 0.000 |

| **Taxonomic level** | | | **Relative abundance in *G. max* "Seed Accessions" (%)** | | | | |  |
| --- | --- | --- | --- | --- | --- | --- | --- | --- |
|  |  |  |  |  |  |  |  |  |
| **Phylum** | **Family** | **Genus** | **AGG-Batch-2** | **Green Harvest** | **AGG-Batch-1** | **Seed Grass** | **Wholesome Supplies** |  |
| *Actinobacteria* | *Actinobacteria* | *Curtobacterium* | **0.889** | 0.016 | 0.000 | 0.039 | **1.955** |  |
| *Actinobacteria* | *Actinobacteria* | *Microbacterium* | 0.000 | 0.000 | **0.359** | 0.078 | 0.000 |  |
| *Actinobacteria* | *Actinobacteria* | Unclassified | 0.058 | 0.000 | 0.016 | 0.000 | **0.107** |  |
| *Actinobacteria* | *Actinobacteria* | *Cutibacterium* | 0.000 | 0.000 | 0.008 | 0.000 | 0.000 |  |
| *Actinobacteria* | *Actinobacteria* | *Amycolatopsis* | 0.000 | 0.000 | 0.008 | 0.000 | 0.000 |  |
| *Bacteroidetes* | *Bacteroidia* | *Hymenobacter* | 0.072 | 0.000 | 0.000 | **0.172** | 0.000 |  |
| *Bacteroidetes* | *Bacteroidia* | *Siphonobacter* | **0.221** | 0.000 | **4.609** | **4.266** | 0.080 |  |
| *Bacteroidetes* | *Bacteroidia* | *Spirosoma* | **0.293** | **1.031** | **0.109** | **0.523** | 0.000 |  |
| *Bacteroidetes* | *Bacteroidia* | *Chryseobacterium* | 0.000 | 0.000 | 0.000 | **3.523** | 0.000 |  |
| *Bacteroidetes* | *Bacteroidia* | *Mucilaginibacter* | **0.841** | 0.000 | **4.195** | **3.383** | 0.000 |  |
| *Bacteroidetes* | *Bacteroidia* | Unclassified | 0.005 | 0.000 | 0.000 | 0.000 | 0.000 |  |
| *Firmicutes* | *Bacilli* | *Bacillus* | **2.543** | **11.203** | **5.820** | **0.898** | **3.366** |  |
| *Firmicutes* | *Bacilli* | *Staphylococcus* | 0.005 | 0.000 | 0.000 | 0.000 | **0.152** |  |
| *Firmicutes* | *Bacilli* | *Carnobacterium* | 0.019 | 0.063 | 0.000 | 0.000 | 0.071 |  |
| *Firmicutes* | *Bacilli* | *Enterococcus* | 0.000 | 0.000 | 0.000 | 0.000 | 0.027 |  |
| *Firmicutes* | *Bacilli* | *Lactobacillus* | 0.005 | 0.016 | 0.000 | 0.000 | 0.071 |  |
| *Proteobacteria* | *Alphaproteobacteria* | *Roseomonas* | 0.087 | 0.000 | **0.586** | **0.172** | 0.000 |  |
| *Proteobacteria* | *Alphaproteobacteria* | *Asticcacaulis* | 0.000 | 0.000 | 0.008 | 0.000 | 0.000 |  |
| *Proteobacteria* | *Alphaproteobacteria* | *Dongia* | 0.000 | 0.000 | 0.008 | 0.000 | 0.000 |  |
| *Proteobacteria* | *Alphaproteobacteria* | *Methylobacterium* | **0.260** | **0.875** | **0.344** | **0.289** | **0.321** |  |
| *Proteobacteria* | *Alphaproteobacteria* | *Microvirga* | 0.000 | 0.000 | 0.070 | 0.000 | 0.000 |  |
| *Proteobacteria* | *Alphaproteobacteria* | *Allorhizobium-Neorhizobium-Pararhizobium-Rhizobium* | 0.000 | 0.047 | **11.320** | 0.063 | 0.000 |  |
| *Proteobacteria* | *Alphaproteobacteria* | Unclassified | 0.000 | 0.000 | 0.016 | 0.039 | 0.000 |  |
| *Proteobacteria* | *Alphaproteobacteria* | *Bradyrhizobium* | 0.005 | 0.000 | 0.000 | 0.000 | 0.036 |  |
| *Proteobacteria* | *Alphaproteobacteria* | Unclassified | 0.000 | 0.000 | 0.008 | 0.000 | 0.000 |  |
| *Proteobacteria* | *Alphaproteobacteria* | *Ferrovibrio* | 0.000 | 0.000 | 0.016 | 0.000 | 0.000 |  |
| *Proteobacteria* | *Alphaproteobacteria* | *Novosphingobium* | **1.341** | 0.000 | **0.430** | **0.672** | 0.009 |  |
| *Proteobacteria* | *Alphaproteobacteria* | *Sphingomonas* | **2.303** | **1.828** | **8.813** | **2.078** | **1.438** |  |
| *Proteobacteria* | *Gammaproteobacteria* | *Achromobacter* | 0.000 | 0.000 | 0.000 | **0.258** | 0.000 |  |
| *Proteobacteria* | *Gammaproteobacteria* | *Burkholderia-Caballeronia-Paraburkholderia* | **0.529** | 0.000 | 0.000 | 0.000 | 0.045 |  |
| *Proteobacteria* | *Gammaproteobacteria* | *Castellaniella* | 0.000 | 0.000 | 0.000 | 0.000 | 0.018 |  |
| *Proteobacteria* | *Gammaproteobacteria* | *Delftia* | 0.000 | 0.000 | 0.000 | **0.297** | 0.063 |  |
| *Proteobacteria* | *Gammaproteobacteria* | *Herbaspirillum* | 0.000 | 0.000 | 0.000 | 0.063 | 0.000 |  |
| *Proteobacteria* | *Gammaproteobacteria* | *Massilia* | **1.582** | **0.844** | **6.344** | **1.484** | 0.098 |  |
| *Proteobacteria* | *Gammaproteobacteria* | *Pandoraea* | 0.000 | 0.000 | 0.008 | 0.000 | 0.000 |  |
| *Proteobacteria* | *Gammaproteobacteria* | *Ralstonia* | 0.063 | 0.000 | 0.000 | 0.039 | 0.000 |  |
| *Proteobacteria* | *Gammaproteobacteria* | Unclassified | 0.029 | 0.016 | **0.156** | **0.531** | 0.098 |  |
| *Proteobacteria* | *Gammaproteobacteria* | Unclassified | 0.000 | 0.000 | 0.008 | 0.000 | 0.000 |  |
| *Proteobacteria* | *Gammaproteobacteria* | *Escherichia-Shigella* | 0.000 | 0.094 | 0.000 | 0.000 | 0.000 |  |
| *Proteobacteria* | *Gammaproteobacteria* | *Pantoea* | **35.745** | **0.500** | 0.016 | **29.695** | **78.170** |  |
| *Proteobacteria* | *Gammaproteobacteria* | Unclassified | 0.058 | **14.078** | 0.063 | 0.047 | 0.000 |  |
| *Proteobacteria* | *Gammaproteobacteria* | *Acinetobacter* | 0.000 | 0.000 | 0.016 | 0.000 | 0.000 |  |
| *Proteobacteria* | *Gammaproteobacteria* | *Pseudomonas* | **53.048** | **65.000** | **56.625** | **51.359** | **13.875** |  |
| *Proteobacteria* | *Gammaproteobacteria* | *Stenotrophomonas* | 0.000 | **4.391** | 0.023 | 0.031 | 0.000 |  |

**Table S7** The relative abundance of bacterial genera associated with different *G. max* seed accessions. Taxa occurring at >0.1% are highlighted in bold.

**Table S8** Closest taxonomy ID identified by Kraken2 for the isolated bacterial and fungal sequences with >96% similarity along with the source of seed accessions. Genome sequences for fungal and bacterial isolates are described under the NCBI BioProjectID PRJNA807720 and PRJNA807698.

| **Closest taxonomy ID identified by Kraken2 for bacterial and fungal sequences{Quast, 2013 #367}** | | | |  |
| --- | --- | --- | --- | --- |
| **Isolate. No** | **Class** | **BLAST hit** | **Seed Accession Source** |  |
| 1 | ***Gammaproteobacteria*** | *Pantoea* | *G. max*_ Seedgrass |  |
| 2 |  | *Pantoea* | *G. max*_ Seedgrass |  |
| 3 |  | *Pantoea* | *G. max*_ Seedgrass |  |
| 4 |  | *Pantoea* | *G. max*_ Seedgrass |  |
| 5 |  | *Pantoea* | *G. max*_ Seedgrass |  |
|  |  |  |  |  |
| 6 |  | *Pseudomonas* | *G. max*_ Seedgrass |  |
|  |  |  |  |  |
| 7 |  | *Pseudomonas* | *G. max*_ Seedgrass |  |
| 8 | ***Alphaproteobacteria*** | *Sphingomonas* | *G. clandestina*_Dandenong |  |
| 9 |  | *Sphingomonas* | *G. clandestina*_Cardinia |  |
| 10 |  | *Sphingomonas* | *G. clandestina*_Wandin |  |
| 11 |  | *Sphingomonas* | *G. clandestina*_Dandenong |  |
|  |  |  |  |  |
|  |  |  |  |  |
|  |  |  |  |  |
| 12 |  | *Sphingomonas* | *G. max*_ Seedgrass |  |
| 13 |  | *Sphingomonas* | *G. max*_ Seedgrass |  |
| 14 |  | *Sphingomonas* | *G. clandestina*_Dandenong |  |
| 15 |  | *Sphingomonas* | *G. clandestina*_Cardinia |  |
| 16 |  | *Methylobacterium* | *G. clandestina*_Dandenong |  |
| 17 |  | *Methylobacterium* | *G. clandestina*_Dandenong |  |
| 18 |  | *Methylobacterium* | *G. clandestina*_Dandenong |  |
|  |  |  |  |  |
| 19 | ***Actinobacteria*** | *Curtobacterium* | *G. clandestina*_Dandenong |  |
| 20 |  | *Curtobacterium* | *G. clandestina*_Dandenong |  |
| 21 |  | *Curtobacterium* | *G. clandestina*_Dandenong |  |
|  |  |  |  |  |
|  |  |  |  |  |
| 22 |  | *Streptomyces* | *G. clandestina*_Wandin |  |
| 23 | ***Bacilli*** | *Bacillus* | *G. clandestina*_Cardinia |  |
| 24 |  | *Bacillus* | *G. max*_Seedgrass |  |
| 25 | ***Flavobacteria*** | *Chryseobacterium* | *G. max*_Seedgrass |  |
| 26 |  | *Chryseobacterium* | *G. max*_Seedgrass |  |
| 27 |  | *Chryseobacterium* | *G. max*_Seedgrass |  |
| 28 | ***Sordariomycetes*** | *Fusarium* | *G. clandestina*_Running creek |  |
| 29 | ***Basidiomycetes*** | *Cryptococcus* | *G. clandestina*_Mornington |  |
| 30 |  | *Cryptococcus* | *G. clandestina*_Butterfield |  |
| 31 |  | *Cryptococcus* | *G. clandestina*_Wandin |  |
| 32 |  | *Cryptococcus* | *G. clandestina*_Running creek |  |
| 33 |  | *Cryptococcus* | *G. clandestina*_Butterfield |  |
| 34 |  | *Cryptococcus* | *G. clandestina*_Wandin |  |
| 35 |  | *Cryptococcus* | *G. clandestina*_Wandin |  |
| 36 |  | *Cryptococcus* | *G. clandestina*_Butterfield |  |


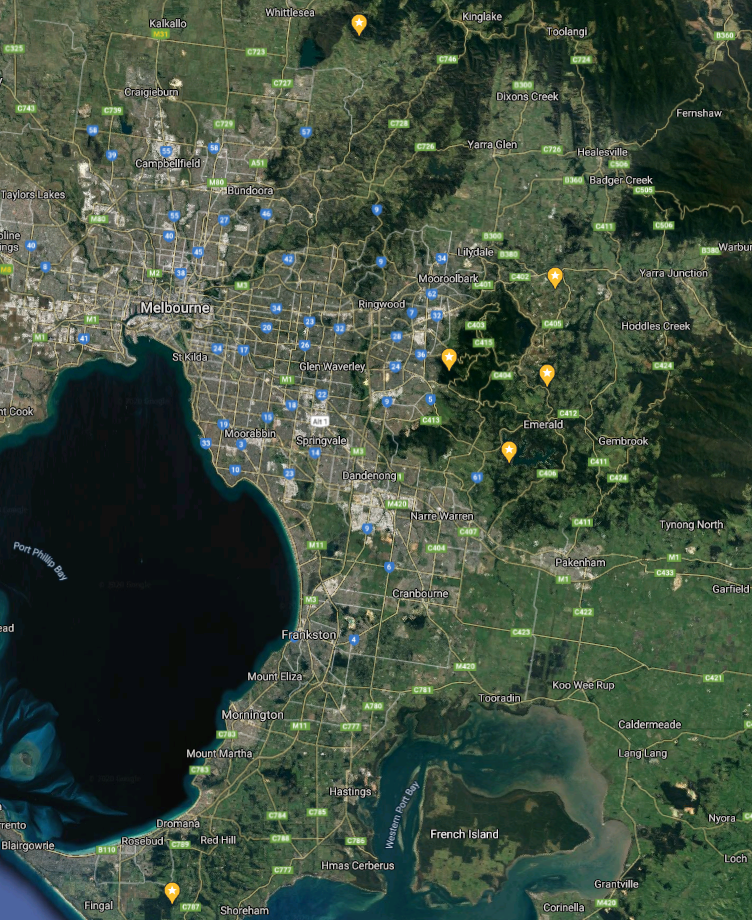


**Figure S1** Map highlighting the sites (in yellow) selected for G. clandestina seed collection in the Greater Melbourne region, Victoria.


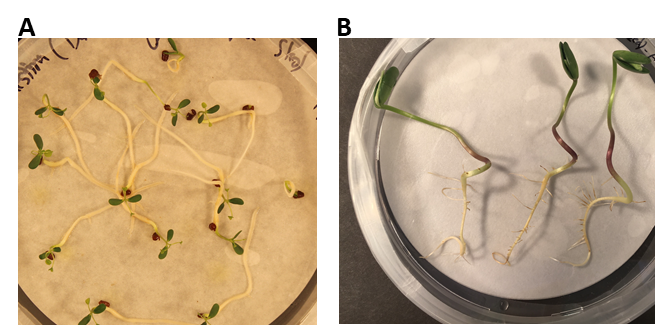


**Figure S2** G. clandestina (A) and G. max (B) seedlings at the unfolded cotyledon growth stage.


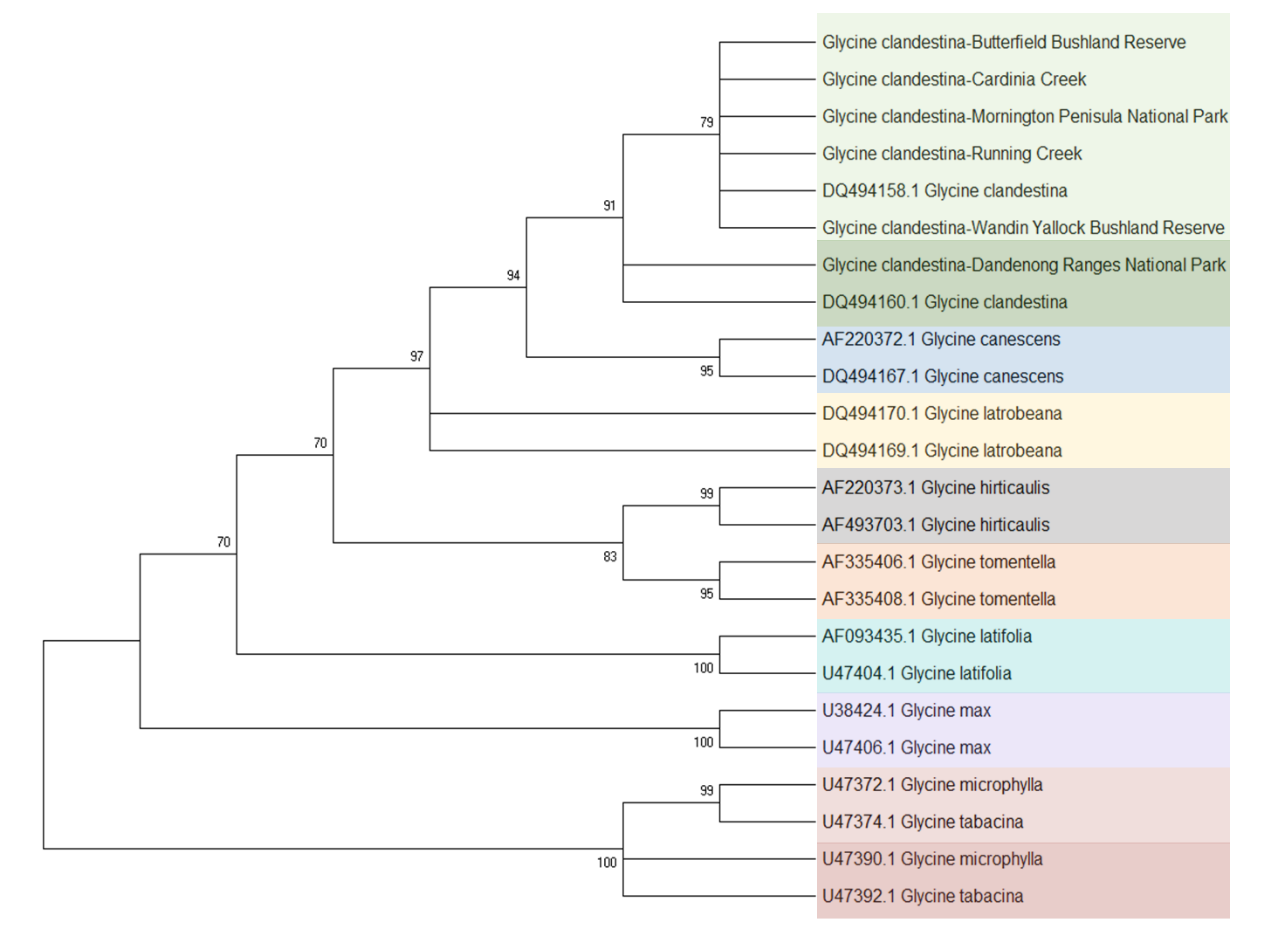


**Figure S3** Maximum Likelihood Consensus Tree with a bootstrap node support of 70% was inferred from the histone H3-D gene sequences of six Glycine taxa used in this study and the 17 references sequences retrieved from NCBI using MEGA X with 1,000 bootstrap replications. The best nucleotide substitution model, the Tamura-Nei model, was used. The ten phylogenetic clades identified within the tree are highlighted in different shades. Sequences reading down (light green, dark green, light blue, yellow, grey, orange, sky blue, violet red and dark red) belong to clades 1 through to 10, respectively.


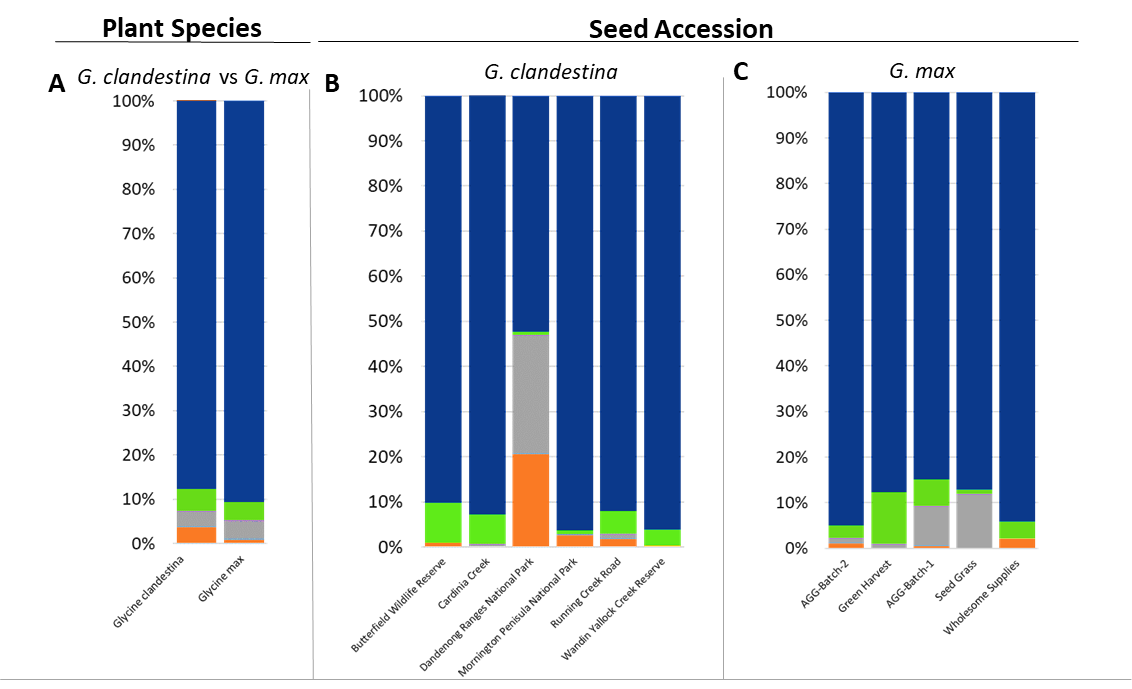

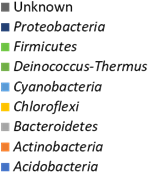


**Figure S4** The relative abundance of bacterial communities across Glycine seeds at phylum level based on “Plant Species” (A), and “Seed Accession” (B, C).

**References**

1. Doyle JJ, Doyle JL, Brown A, Pfeil BE: **Confirmation of shared and divergent genomes in the G. tabacina polyploid complex (Leguminosae) using histone H3-D sequences**. *Systematic Botany* 2000:437-448.

2. Pfeil B, Craven L, Brown A, Murray B, Doyle J: **Three new species of northern Australian G. (Fabaceae, Phaseolae), G. gracei, G. montis-douglas and G. syndetika**. *Australian Systematic Botany* 2006, **19**(3):245-258.

3. Doyle JJ, Doyle JL, Brown A, Palmer RG: **Genomes, multiple origins, and lineage recombination in the G. tomentella (Leguminosae) polyploid complex: histone H3‐D gene sequences**. *Evolution; International Journal of Organic Evolution* 2002, **56**(7):1388-1402.

4. Doyle JJ, Kanazin V, Shoemaker RC: **Phylogenetic utility of histone H3 intron sequences in the perennial relatives of soybean (G.: Leguminosae)**. *Molecular Phylogenetics and Evolution* 1996, **6**(3):438-447.

5. Doyle JJ, Doyle JL, Brown A: **Incongruence in the diploid B-genome species complex of G. (Leguminosae) revisited: histone H3-D alleles versus chloroplast haplotypes**. *Molecular Biology and Evolution* 1999, **16**(3):354-362.

6. Kanazin V, Blake T, Shoemaker RC: **Organization of the histone H3 genes in soybean, barley and wheat**. *Molecular and General Genetics MGG* 1996, **250**(2):137-147.

7. Brown A, Doyle J, Grace J, Doyle J: **Molecular phylogenetic relationships within and among diploid races of G. tomentella (Leguminosae)**. *Australian Systematic Botany* 2002, **15**(1):37-47.
