# Supplementary material for: Australian native Glycine clandestina seed microbiota hosts a more diverse bacterial community than the domesticated soybean Glycine max
Source: Environ Microbiome. 2022 Nov 16;17:56. doi: 10.1186/s40793-022-00452-y (PMC9670509; doi:10.1186/s40793-022-00452-y)
Supplement: Supplementary file 2 — Additional file 2. Figure S1. MALDI-TOF MS spectra generated from a subset of bacteria isolated from G. clandestina and G. max seeds. The bacterial isolates were identified using an in-house endophyte library and Bruker database. The isolates selected for WGS are labelled next to the representative clade. [file 40793_2022_452_MOESM2_ESM.pdf]

Additional file 2:

Australian native *Glycine clandestina* seed microbiota host more diverse bacterial comm unities than the domesticated soybean *Glycine max*

Ankush Chandel[1,2*], Ross Mann[1], Jatinder Kaur[1], Ian Tannenbaum[1], Sally Norton[3], Jacqueline Edwards[1,2], German Spangenberg[1,2], Tim Sawbridge[1,2]

Additional file 2 contains:

Figure S1

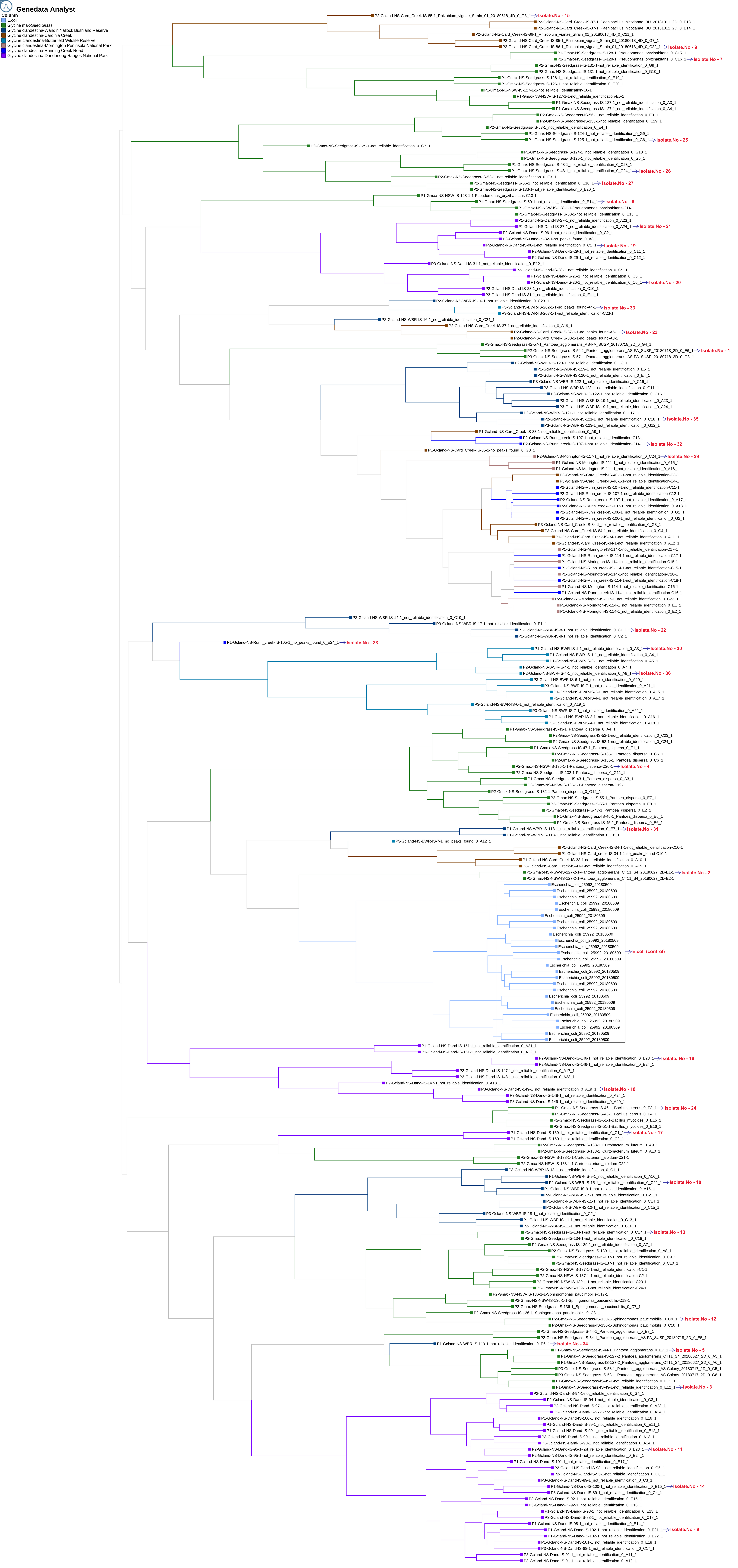
Genedata Analyst
Column
E.coli
Glycine max-Seed Grass
Glycine clandestina-Wandin Yallock Bushland Reserve
Glycine clandestina-Cardinia Creek
Glycine clandestina-Butterfield Wildlife Reserve
Glycine clandestina-Mornington Peninsula National Park
Glycine clandestina-Running Creek Road
Glycine clandestina-Dandenong Ranges National Park
E.coli (control)

Figure S1 MALDI-TOF MS spectra generated from a subset of bacteria isolated from *G. clandestina* and *G. max* seeds. The bacterial isolates were identified using an in-house endophyte library and Bruker database. The isolates selected for WGS are labelled next to the representative clade.
